# Straightforward Regio- and Diastereoselective Synthesis, Molecular Structure, Intermolecular Interactions and Mechanistic Study of Spirooxindole-Engrafted Rhodanine Analogs

**DOI:** 10.3390/molecules26237276

**Published:** 2021-11-30

**Authors:** Assem Barakat, Matti Haukka, Saied M. Soliman, M. Ali, Abdullah Mohammed Al-Majid, Ayman El-Faham, Luis R. Domingo

**Affiliations:** 1 Department of Chemistry, College of Science, King Saud University, Riyadh 11451, Saudi Arabia; maly.c@ksu.edu.sa (M.A.); amajid@ksu.edu.sa (A.M.A.-M.); 2 Department of Chemistry, University of Jyväskylä, FI-40014 Jyväskylä, Finland; matti.o.haukka@jyu.fi; 3 Department of Chemistry, Faculty of Science, Alexandria University, Alexandria 21321, Egypt; saied1soliman@yahoo.com (S.M.S.); ayman.elfaham@alexu.edu.eg (A.E.-F.); 4 Department of Organic Chemistry, University of Valencia, 46100 Burjassot, Spain; domingo@utopia.uv.es

**Keywords:** spirooxindole, rhodanine, azomethine ylides, [3 + 2] cycloaddition (32CA) reactions, hydrogen bonding, Hirshfeld DFT, supernucleophiles

## Abstract

Straightforward regio- and diastereoselective synthesis of bi-spirooxindole-engrafted rhodanine analogs **5a**–**d** were achieved by one-pot multicomponent [3 + 2] cycloaddition (32CA) reaction of stabilized azomethine ylide (AYs **3a**–**d**) generated in situ by condensation of L-thioproline and 6-chloro-isatin with (*E*)-2-(5-(4-chlorobenzylidene)-2,4-dioxothiazolidin-3-yl)-*N*-(2-morpholinoethyl)acetamide. The bi-spirooxindole-engrafted rhodanine analogs were constructed with excellent diastereo- and regioselectivity along with high chemical yield. X-ray crystallographic investigations for hybrid **5a** revealed the presence of four contiguous stereocenters related to C11, C12, C19 and C22 of the spiro structure. Hirshfeld calculations indicated the presence of many short intermolecular contacts such as Cl...C, S...S, S...H, O...H, N...H, H...C, C...C and H...H interactions. These contacts played a very important role in the crystal stability. The polar nature of the 32CA reaction was studied by analysis of the conceptual DFT reactivity indices. Theoretical study of this 32CA reaction indicated that it takes place through a non-concerted *two-stage one-step* mechanism associated with the nucleophilic attack of AY **3a** to the electrophilic ethylene derivative.

## 1. Introduction

Rhodanines′ (2-thioxothiazolidin-4-ones) privileged structure has attracted much attention in pharmaceutical and medicinal chemistry [1,2]. These substituted rhodanines have been discovered and reported for several pharmaceutical applications, including topoisomerase II inhibition potency [3]; potential AChE inhibitors [4,5]; HIV-1 integrase inhibitors [6]; anti-cancer agent towards MCF-7 breast cancer cells [7] and anti-diabetic (T2DM) targeting the inhibition of enzymes such as aldose reductase (ALR) [8], α-glucosidase [9], α-amylase [10] and PTP1B enzyme [11], and other therapeutic targets have also been reported, such as antibacterial ones [12]. Chemical modification, structural elucidation and molecular interactions investigations of these compounds are still of high interest.

On the other hand, one-pot multicomponent [3 + 2] cycloaddition (32CA) reaction has been established as a powerful and efficient approach for stereoselective synthesis of the spirooxindole pyrrolidine core structure in a wide range of natural products and pharmaceutical compounds [13,14,15,16,17,18,19]. The key step of this strategy is the generation of azomethine ylides (AYs) which then react with electron-deficient ethylene to produce pyrrolidine-spirooxindole with contiguous stereocenters. These pyrrolidine-spirooxindole molecules in particular have shown a panoply of significant pharmaceutical targets and applications, such as MDM2 inhibitors [20,21,22], anti-cancer treatment [7,23], AChE inhibitors [24,25,26] and prospective activity against SARS-CoV-2 [27]. Structural modifications have emerged as attractive synthetic targets for researchers.

Several representative examples have been reported in the literature disclosing the spirooxindole scaffold, but combination of the rhodanine motif with the spirooxindole hybrid is still less discussed. Knorr, M. and his team reported the synthesis of rhodanine-substituted spiro[pyrrolidine-oxindole] analogs and exploited this rhodanine type as an anti-diabetic agent via inhibition of the α-amylase enzyme [28]. Recently, the research group headed by El-Faham reported the rhodanine subtype carboxamide with a backbone of several amino acids using OxymaPure/*N*,*N*′-diisopropylcarbodimide coupling methodology and evaluated these compounds for their antimicrobial activities [29].

Inter- or intramolecular hydrogen bonding interactions were found to play a crucial role in different systems which contribute to the conformational stability of proteins [30] and aid in the dimerization or aggregation of the supramolecular chemistry [31].

Recent advances made in the theoretical understanding of 32CA reactions based on the Molecular Electron Density Theory [32] (MEDT) have allowed for establishing a very good correlation between the electronic structure of the simplest three-atom-components (TACs) and their reactivity towards ethylene [33]. The simplest AY, CH_2_-NH-CH_2_, is a very reactive *pseudodiradical* TAC participating in *pdr-type* 32CA reactions without any appreciable barrier [34]. However, substitution on the experimental TACs stabilizes them, thus changing the electronic structure and, consequently, the reactivity to that of a *zw-type* 32CA reaction [35]. Consequently, this type of 32CA reaction demands adequate nucleophilic/electrophilic activation of the reagents to take place [35]. Synthesis of a new ethylene system and its employment for 32CA reaction for the purpose of theoretical study understanding by MEDT are a challenge.

**Scheme 1:**
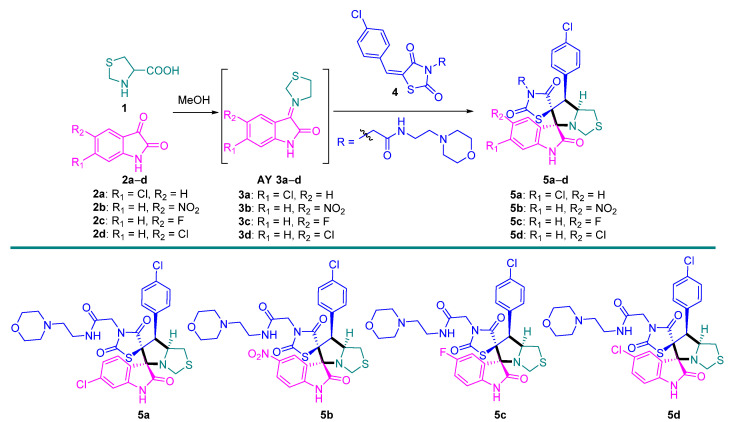
Synthesis of compounds **5a**–**d** via 32CA reaction of AYs **3a**–**d** with ethylene derivative **4**.

Based on the findings above and continuing in the contribution to the synthesis of new spiro-heterocycle hybrids, in this study we report the straightforward synthesis of rhodanine-substituted spiro[pyrrolidine-oxindole]-carboxamide/morpholine derivatives **5a**–**d** using the 32CA reaction methodology. Thermal prototropy generated the stabilized AYs **3a**–**d** in situ, which then reacted with the rhodanine subtype **4** to produce the desired compounds **5a**–**d** (see Scheme 1). Furthermore, molecular structure and intermolecular interactions, the X-ray crystal structure of **5a**, Hirshfeld analysis and conceptual DFT studies at the ground state of the reagents are also discussed.

## 2. Results and Discussion

**Scheme 2:**
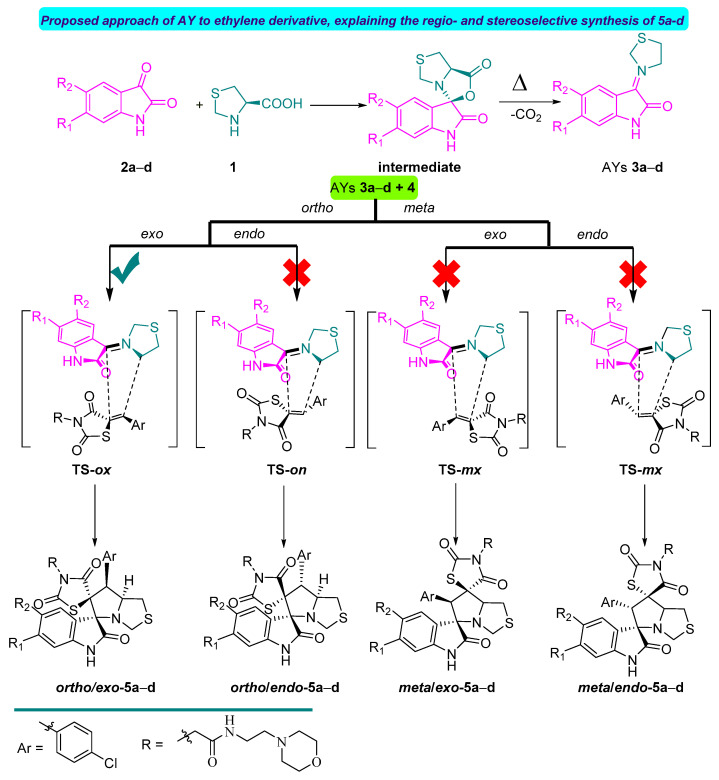
Synthesis and plausible mechanism of the desired spirooxindole derivatives **5a**–**d**.

The straightforward regio- and diastereoselective synthesis of the desired bi-spirooxindole-engrafted rhodanine analogs **5a**–**d** is shown in Scheme 1. The requisite starting material named (*E*)-2-(5-(4-chlorobenzylidene)-2,4-dioxothiazolidin-3-yl)-*N*-(2-morpholinoethyl)acetamide **4** was synthesized following the method reported in the literature [29]. Employing the one-pot multicomponent-based 32CA reaction approach of the arylidene rhodanine analog **4** with di-carbonyl compounds (6-chloroisatin **2a**; 5-nitroisatin **2b**; 5-fluoroisatin **2c**; 5-chloroisatin **2d**) and thioproline **1** under reflux conditions in MeOH for 2 h produced the target stereoselective bi-spirooxindole-engrafted rhodanine analogs **5a**–**d**. The reaction proceeded initially with isatins **2a**–**d** reacting with the secondary amino acid (L-thioproline) **1** to generate the azomethine ylides in situ (AYs **3a**–**d**). In the second step, the generated azomethine ylides (AYs **3a**–**d**) reacted with the arylidene rhodanine analog **4** with four possible approach modes: two regioisomeric approach modes of the aryl-substituted carbon of **4** to the two carbons of the azomethine ylides (AYs) framework of AYs **3a**–**d**, named *ortho* and *meta*, and two stereoisomeric approach modes of the C–C double bond of **4** to AYs **3a**–**d**, named *endo* and *exo*. The four possible alternative reaction paths are shown in Scheme 2. The formation of the bi-spirooxindole-engrafted rhodanine analogs **5a**–**d** indicates that this 32CA reaction is completely *ortho* regioselective and *exo* stereoselective, taking place via the transition state structure TS-ox. The spectral data of the bi-spirooxindole-engrafted rhodanine analogs **5a**–**d** were assigned and elucidated and were found to be in good agreement with the proposed structure. The chemical structure of **5a** was further confirmed by single-crystal X-ray diffraction analysis.

### 2.1. Crystal Structure Description

**Figure 1:**
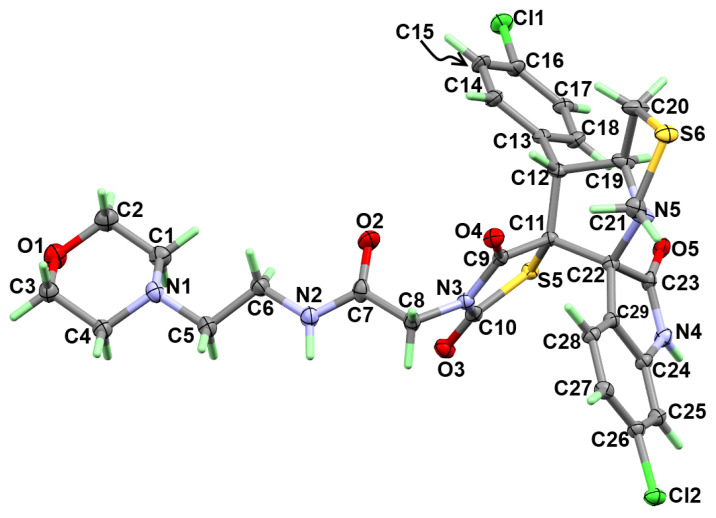
Atom numbering and thermal ellipsoids at 30% probability level for **5a**. Only one unit (unit a) is presented; the second unit and the crystallized solvent molecules are not shown for simplicity.

The X-ray structure of **5a** showing atom numbering and thermal ellipsoids drawn at the 30% probability level is shown in Figure 1. The structure agrees very well with the spectral analyses. The crystal data and structure refinement details are depicted in Table 1, while selected bond distances and angles are listed in Table 2. The compound crystallized in the monoclinic system and *P2**_1_*** space group with unit cell parameters of *a* = 14.0397(2) Å, *b* = 15.9918(2) Å, *c* = 14.1488(2) Å and *β* = 109.439(2)°. The unit cell volume was 2995.61(8) Å^3^ and the number of molecules per unit cell was 4. The asymmetric unit comprised two crystallographically independent molecules of **5a** and two water molecules. The geometrical parameters of the two units are marginally different. The reported X-ray structure revealed very well the presence of four asymmetric carbon centers in the spiro-system, namely C11, C12, C19 and C22 (Figure 1).

**Figure 2:**
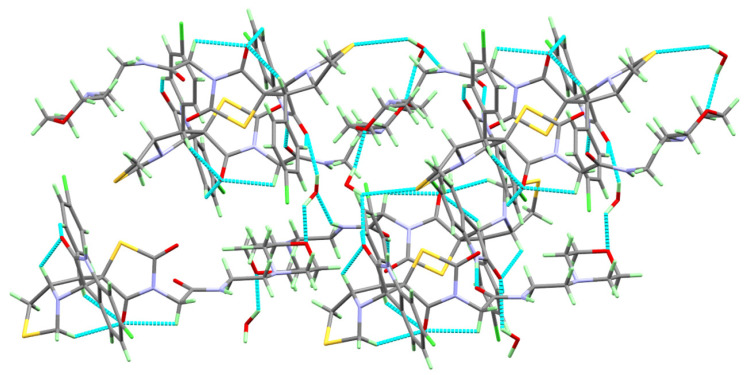
Packing of molecular units via hydrogen bonding interactions in compound **5a**.

The molecules of **5a** are connected with each other, including the solvent water, via a complicated set of strong N-H...O, O-H...O and O-H...S hydrogen bonding interactions as well as weak C-H...O interactions (Table 3). The hydrogen bond network is shown in Figure 2.

### 2.2. Analysis of Molecular Packing

In light of the great importance of intermolecular interactions for crystal stability, Hirshfeld calculations were employed in order to analyze the molecular packing in the studied system. The Hirshfeld surfaces of **5a** are shown in Figure S1 (Supplementary Materials). The crystal structure of **5** indicated the presence of two molecules as an asymmetric unit; hence, the Hirshfeld surfaces of both units are presented in this figure.

**Figure 3:**
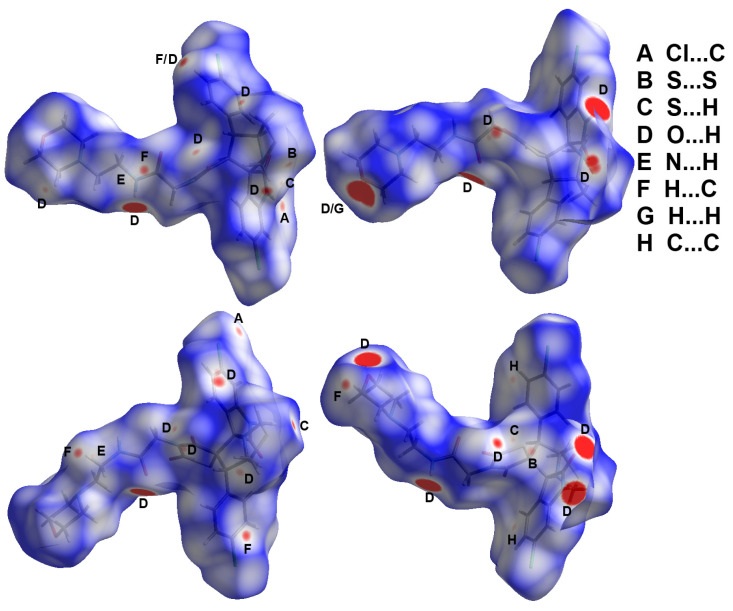
The d_norm_ maps of the two molecular units (top: unit A; bottom: unit B) showing the most important contacts in **5a**.

In Figure 3, the d_norm_ maps of both molecules are also presented. The red regions indicate the atomic sites included in strong intermolecular interactions with the neighboring molecules. It is clear that the molecular packing in the studied system is controlled by many short contacts such as Cl...C, S...S, S...H, O...H, N...H, H...C, C...C and H...H interactions.

**Figure 4:**
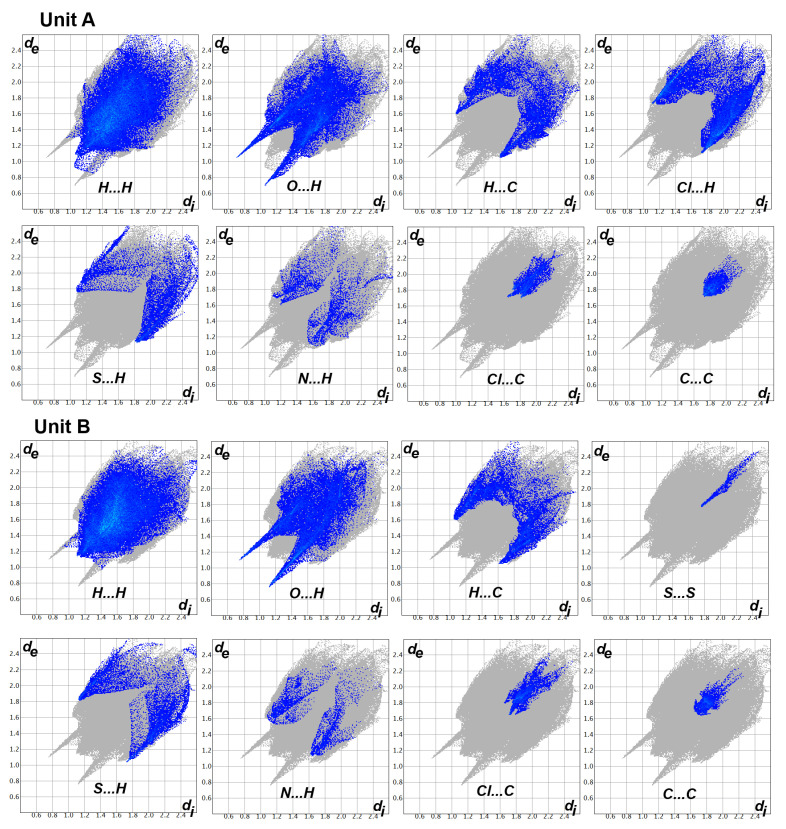
Decomposed fingerprint plots of the most dominant contacts of **5a**.

**Figure 5:**
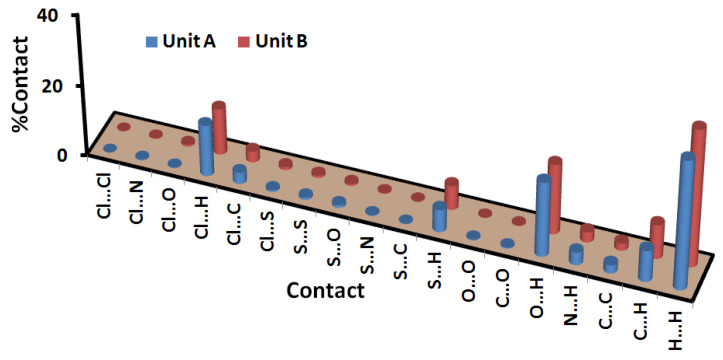
The percentages of all possible intermolecular interactions in the crystal structure of **5a**.

In addition, fingerprint plots gave a quantitative summary for all intermolecular interactions. Decomposed fingerprint plots enabled us to calculate the percentage of each intermolecular interaction (Figure 4). The distribution of all possible intermolecular interactions is shown in Figure 5. It is clear that the H...H (38.8%), O...H (19.9%), Cl...H (13.1%) and H...C (9.7%) contacts are the most dominant in molecular unit B in the atom numbering of the heavy atoms (unit B). The results for the other molecular unit (unit A) are almost the same (Table S1, Supplementary Materials).

Both the fingerprint plots and d_norm_ maps provide clear evidence on the short intermolecular interactions which control the crystal stability. Many short O...H interactions occurred in this crystal. A summary of the most important contacts and the corresponding interaction distances is listed in Table 4. The O5B…H1SB (1.974 Å), O2…H5 (1.765 Å), O2B…H5BA (1.941 Å), O2S…H2BA (1.856 Å), O1B…H2S (1.886 Å) and O1S…H2 (1.995 Å) hydrogen bonds are significantly short and hence have great importance in the molecular packing. The two spikes in the decomposed fingerprint plot of the O...H interactions indicate not only strong hydrogen bonds but also the molecule acting as both the hydrogen bond donor and acceptor. In the same table, all contacts with a distance shorter than the vdWs radii sum of the interacting atoms are also presented. The only exception is the N2…H6BA (2.683Å) contact, which has a slightly longer contact distance than the vdWs radii sum of the N and H atoms (2.64 Å).

### 2.3. DFT Studies of the Structure of ***5a***

**Figure 6:**
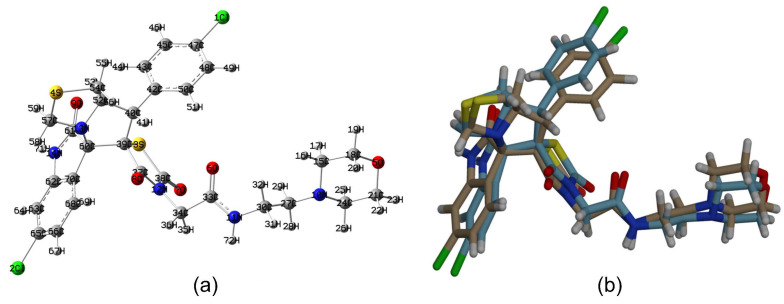
The optimized geometry (**a**) and overlay of the optimized geometry with experimental structures (**b**)for **5a**.

**Figure 7:**
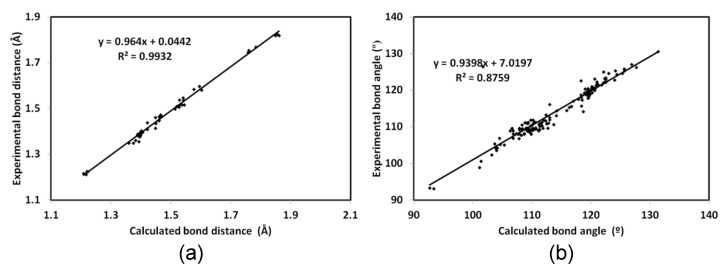
Correlations between the calculated and experimental (**a**) bond distances, and (**b**) bond angles.

The molecular geometry of **5** was calculated and the resulting optimized structure is shown in Figure 6 (right part). As can be seen from Table 5, there are good agreements between the calculated and experimental bond distances. In addition, there are straight-line correlations between the calculated and experimental bond distances (R^2^ = 0.9932) and angles (R^2^ = 0.8759), as shown in Figure 7. In addition, the structure matching between the calculated and experimental structures is shown in Figure 6 (left part). Generally, the structural parameters indicate good agreement between the calculated results and X-ray geometry, with some little variations which could be attributed to the packing effects.

**Figure 8:**
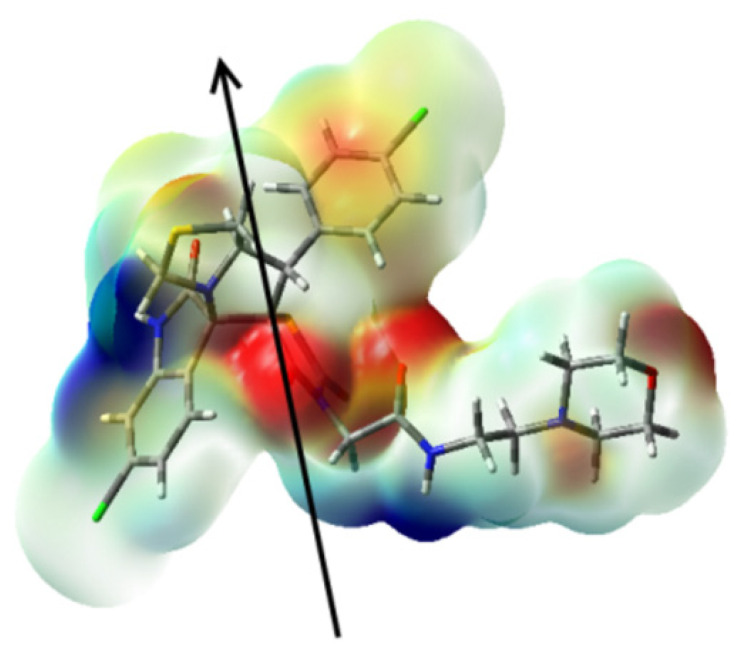
The MEP of **5a**.

The charges at the different atoms of **5** are collated in Table 6. The results indicate the presence of a number of negatively charged atomic sites, including N, O and the majority of C sites. There are five oxygen atoms and five nitrogen sites in **5a** where the *exo* carbonyl oxygen and the neighboring amidic nitrogen atoms are the most negative, respectively. All carbon atoms bonded to N or O atoms have highly positive natural charges. In addition, the NH protons are the most positive hydrogen atoms (0.4475 and 0.4196 e). These results are in good agreement with the molecular electrostatic potential map (MEP) shown in Figure 8. In the MEP, the red colored areas are related to regions of high electron density while the blue colored areas are related to highly electron-deficient regions. The red regions are related to carbonyl oxygen while the blue regions are close to the NH protons. The studied compound has a polar nature with a net dipole moment of 2.5735 Debye, and the direction of the dipole moment vector is shown in Figure 8.

### 2.4. Conceptual DFT Analysis of the 32CA Reaction of AYs ***3a–d*** with Ethylene Derivative ***4***

The reactivity indices defined within the conceptual DFT (CDFT) [36,37] have been shown to be powerful tools to understand the reactivity in polar reactions. The global reactivity indices, namely the electronic chemical potential *μ*, chemical hardness *η*, global electrophilicity ω and global nucleophilicity *N*, for AYs **3a**–**d** and ethylene **4** involved in this 32CA reaction and the reduced models AY **6** and ethylene **7** are gathered in Table 7.

The electronic chemical potentials [36] *μ* of AYs **3a**–**d**, ranking from *μ* = −3.26 (**3c**) to −3.71 (**3b**) eV, are higher than that of the ethylene derivative **4**, *μ* = −4.30 eV, indicating that along a polar 32CA reaction, the global electron density transfer (GEDT) [38] will take place from AYs **3a**–**d** to the ethylene derivative **4**, with the 32CA reaction being classified as the forward electron density flux (FEDF) [39].

The electrophilicity index ω [40] of AYs **3a**–**d** ranges from 1.62 (**3c**) to 2.14 (**2b**) eV, thus being classified as strong electrophiles within the electrophilicity scale [37]. On the other hand, AYs **3a**–**d** present a nucleophilicity index *N* [41] between 3.79 (**3d**) and 4.23 (**3c**) eV, thus being classified as strong nucleophiles within the nucleophilicity scale [37]. The strong nucleophilic character of AYs **3a**, **3c** and **3d**, higher than 4.0 eV, allows for their classification as supernucleophiles [42]. Note that the presence of the strong electron-withdrawing NO_2_ group in AY **3b** markedly increases its electrophilicity index ω to 2.14 eV and decreases its nucleophilicity index *N* to 3.79 eV. In spite of these behaviors, AY **3b** is a very strong nucleophile participating in polar 32CA reactions of FEDF.

The ethylene derivative **4** presents an electrophilicity index ω of 2.51 eV, being classified as a strong electrophile within the electrophilicity scale. On the other hand, it presents a nucleophilicity index *N* of 3.09 eV, being classified also as a strong nucleophile within the nucleophilicity scale.

The similar electrophilicity ω and nucleophilicity *N* indices of the reduced models AY **6** and ethylene **7** to those of the experimental AYs **3a**–**d** and ethylene **4** (see Table 7) indicate that the corresponding 32CA reactions will be very similar.

The highly nucleophilic character of AYs **3a**–**d** and **6** together with the strong electrophilic character of the ethylene derivatives **4** and **7** indicate that the corresponding 32CA reactions will have a highly polar character, being classified as FEDF [39].

**Figure 9:**
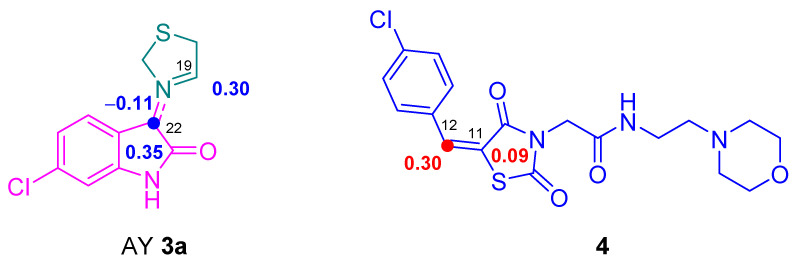
B3LYP/6-31G(d) nucleophilic Parr functions, in blue, of AY **3a**, and electrophilic Parr functions, in red, of the ethylene derivative **4**.

In a polar reaction involving non-symmetric species, the most favorable reaction path involves the two-center interaction between the most electrophilic and the most nucleophilic centers [43]. Many studies have shown that analysis of the electrophilic P_k_^+^ and nucleophilic P_k_^−^ Parr functions [44], resulting from the excess of spin electron density gathered via the GEDT [38], is one of the most accurate and insightful tools for the analysis of the local reactivity in polar and ionic processes. Hence, according to the characteristics of the reagents, the P_k_^−^ nucleophilic Parr functions of AY **3a** and the P_k_^+^ electrophilic Parr functions of the ethylene derivative **4** were analyzed (see Figure 9).

The two carbons of AY **3a** are nucleophilically activated by P_k_^−^ = 0.35 (C22) and 0.30 (C19), the exocyclic C22 carbon being slightly more activated. Note that the N5 nitrogen is deactivated by P_k_^−^ = −0.11. On the other hand, the conjugated C12 carbon of the ethylene derivative **4** is electrophilically activated, P_k_^+^ = 0.30, while the exocyclic C11 carbon in marginally electrophilically activated, P_k_^+^ = 0.09.

Analysis of the Parr functions suggest that the *meta* reaction paths will be slightly more favorable than the *ortho* ones. However, steric hindrances present along then *meta* reaction paths will be responsible of the meta regioselectivity experimentally observed. These steric hindrances can also explain that the *ortho/exo* reaction path will be favored with respect to the *ortho/endo* one.

### 2.5. Study of the Reaction Mechanism

**Scheme 3:**
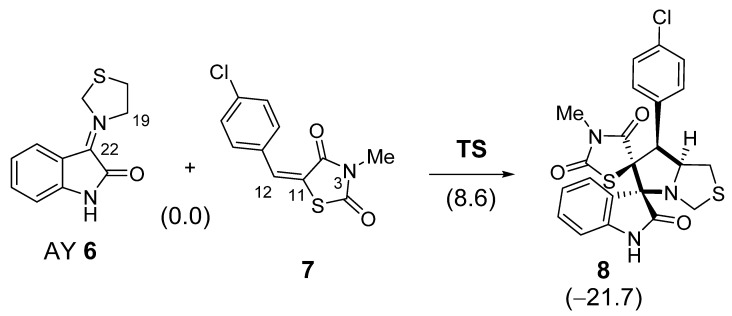
The 32CA reaction of AY **6** with the ethylene derivative **7**. Relative energies, with respect to separated reagents, are indicated in parentheses in kcal·mol^−1^.

For the study of the mechanism of the 32CA reactions of AYs **3a**–**d** with ethylene **6**, a reduced model was selected (see Scheme 3). In this reaction model, the substituents present at the aromatic ring of AYs **3a**–**d** were removed, while the chain present at the amide N3 nitrogen of the ethylene derivative **4** was replaced by a methyl group in ethylene **7**. Analysis of the CDFT indices of AY **6** and ethylene **7** showed that they have a similar nucleophilic and electrophilic character compared to the experimental AYs **3a**–**d** and ethylene **4**, indicating that the former will present a similar reactivity (see Table 7). Analysis of the reaction path indicated that this 32CA reaction takes place though a one-step mechanism. The B3LYP/6-31G(d) relative energies are given in Scheme 3. The activation energy associated to this 32CA reaction is 8.6 kcal·mol^−1^, with the reaction being exothermic by −21.7 kcal·mol^−1^.

**Figure 10:**
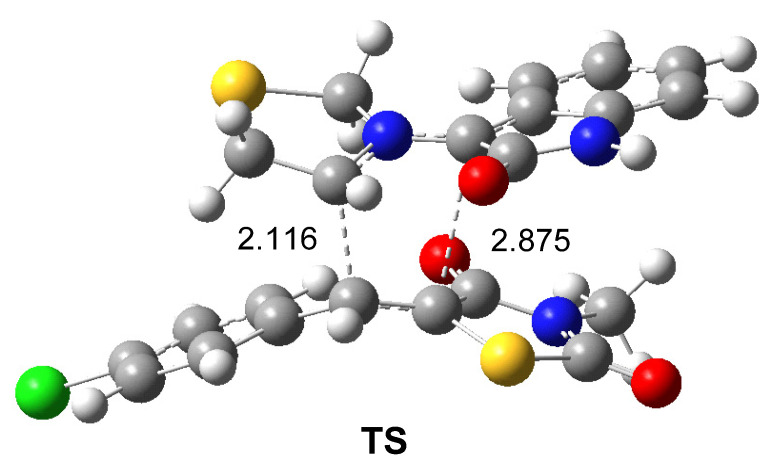
B3LYP/6-31G(d) geometry of TS. The distances are given in Angstrom.

The geometry of the transition state structure **TS** is given in Figure 10. The C–C distances between the two pairs of interacting carbons are 2.116 (C12–C19) and 2.875 (C11–C22) Å. These distances indicate that this TS is associated with a highly asynchronous C–C single-bond formation process, in which the shorter C12–C19 distance corresponds to that involving the most electrophilic carbon C12 of the ethylene derivative **7**. These C–C distances, which are higher than 2.1 Å, indicate that the formation of the two C–C single bonds had not begun at the TS [38]. Analysis of the intrinsic reaction coordinates [45] associated to the highly asynchronous TS indicated that this 32CA reaction takes place through a non-concerted *two-stage one-step* mechanism [46] in which formation of the second C11–C22 single bond begins when the first C12–C19 single bond is completely formed.

Finally, analysis of the GEDT [38] at the TS allowed for assessment of the polar character of this 32CA reaction. GEDT values lower than 0.05 e correspond with non-polar processes, while values higher than 0.20 e correspond with polar processes. The GEDT value at the TS was 0.26 e. This high GEDT value is a consequence of the supernucleophilic character of AY **6** and the strong nucleophilic character of ethylene **7**. The flux of the electron density, which goes from AY **6** to ethylene **7**, classifies this 32CA reaction as FEDF, in clear agreement with the analysis of the CDFT indices.

## 3. Materials and Methods

### 3.1. General Notes

Isatin derivatives **2a**–**d** and thioproline **1** are commercially available (Sigma-Aldrich Chemie GmbH, Riedstr, Germany; and Alfa Aesar GmbH & Co KG, Karlsruhe, Germany). ^1^H-NMR and ^13^C-NMR spectra were recorded in DMSO-*d*_6_ (Jeol Spectrometer (400 MHz), (Jeol, Tokyo, Japan). X-ray diffraction data were collected on a Rigaku Oxford Diffraction Supernova diffractometer and processed with CrysAlisPro software v. 1.171.41.93a (Rigaku Oxford Diffraction, Yarnton, UK, 2020) using Cu Kα radiation.

### 3.2. Synthesis of Compound ***4***

The desired starting material **4** was synthesized according to the reported literature [29].

### 3.3. General Method for the Synthesis of ***5a**–**d***

A mixture of three components including isatin derivatives **2a**–**d** (0.5 mmol), L-thioproline **1** (66.5 mg, 0.5 mmol) and the compound **4** (204.5 mg, 0.5 mmol) was refluxed in an oil bath for 2 h. After completion of the reaction as evident from tlc (TLC Eluent: Ethyl acetate: *n-*Hexane 40%), the reaction mixture was kept at room temperature overnight for slow evaporation, and the solid crystalline materials were filtered off to obtain compounds **5a**–**d** without any further purification as faint yellow solid compounds with 80–90% chemical yield.

2-((3*S*,6′*S*,7′*S*,7a′*S*)-6-chloro-7′-(4-chlorophenyl)-2,2″,4″-trioxo-7′,7a′-dihydro-1′*H*,3′*H*-dispiro[indoline-3,5′-pyrrolo[1,2-*c*]thiazole-6′,5″-thiazolidin]-3″-yl)-*N*-(2-morpholinoethyl)acetamide **5a**

According to the general method, the 6-chloro-isatin **2a** (90.5 mg, 0.5 mmol) was utilized and the final compound **5a** was obtained as a faint yellow solid compound with 85% chemical yield.

^1^H-NMR (400 Hz, DMSO-*d*_6_): *δ* 11.17 (s, 1H, NH), 8.06 (t, *J* = 5.6 Hz, 1H, NHCO), 7.48 (d, *J* = 8.2 Hz, 2H, C_6_H_4_), 7.41 (d, *J* = 8.2 Hz, 2H, C_6_H_4_), 7.26 (d, *J* = 8.2 Hz, 1H, C_6_H_3_ (oxindole)), 7.07 (d, *J* = 10.2 Hz, 1H, C_6_H_3_ (oxindole)), 6.90 (s, 1H, C_6_H_3_ (oxindole)), 4.85 (q, *J* = 6.9 Hz, 1H), 4.18 (d, *J* = 9.4 Hz, 1H), 3.97 (q, *J* = 5.4 Hz, 2H), 3.82 (d, *J* = 6.2 Hz, 1H), 3.56 (d, *J* = 4.5 Hz, 4H), 3.46 (d, *J* = 6.0 Hz, 1H), 3.19–3.13 (m, 2H), 3.02 (q, *J* = 5.5 Hz, 1H), 2.77 (d, *J* = 7.8 Hz, 1H), 2.37–2.30 (m, 6H); ^13^C-NMR (100 Hz, DMSO-*d*_6_): *δ* 176.2, 174.7, 168.7, 164.7, 158.3, 154.7, 151.3, 145.4, 135.9, 135.6, 133.4, 132.3, 131.5, 129.1, 121.6, 110.8, 76.2, 75.5, 70.1, 66.7, 57.7, 55.9, 53.8, 47.1, 43.9, 36.7, 33.3; Chemical Formula: C_29_H_29_Cl_2_N_5_O_5_S_2;_ LCMS (*m*/*z*): 662.18 [M + H]^+^; Elemental Analysis: [Calculated: C, 52.57; H, 4.41; N, 10.57; S, 9.68; Found: C, 52.61; H, 4.40; N, 10.69; S, 9.79]. IR (KBr, cm^−1^): 3424, 3289, 3081, 2928, 2857, 1710, 1620, 1564, 1229, 1139.

2-((3*S*,6′*S*,7′*S*,7a′*S*)-7′-(4-chlorophenyl)-5-nitro-2,2″,4″-trioxo-7′,7a′-dihydro-1′*H*,3′*H*-dispiro[indoline-3,5′-pyrrolo[1,2-*c*]thiazole-6′,5″-thiazolidin]-3″-yl)-*N*-(2-morpholinoethyl)acetamide **5b**

According to the general method, the 5-nitro-isatin **2b** (96 mg, 0.5 mmol) was utilized and the final compound **5b** was obtained as a faint yellow solid compound with 80% chemical yield.

^1^H-NMR (400 Hz, DMSO-*d*_6_): δ 11.70 (s, 1H), 8.32–8.23 (m, 1H), 8.07 (t, *J* = 5.3 Hz, 2H), 7.97 (s, 1H), 7.68 (d, *J* = 8.6 Hz, 1H), 7.62 (d, *J* = 8.6 Hz, 1H), 7.52–7.41 (m, 4H), 7.08 (d, *J* = 8.7 Hz, 1H), 4.79 (q, *J* = 7.8 Hz, 1H), 4.28 (s, 1H), 4.18 (d, *J* = 9.4 Hz, 1H), 4.03 (d, *J* = 16.6 Hz, 1H), 3.93 (d, *J* = 16.3 Hz, 1H), 3.85 (d, *J* = 6.4 Hz, 1H), 3.70 (s, 1H), 3.57 (t, *J* = 5.0 Hz, 6H), 3.47 (d, *J* = 6.2 Hz, 1H), 3.20 (dd, *J* = 13.8, 7.5 Hz, 1H), 3.11–2.98 (m, 3H); ^13^C-NMR (100 Hz, DMSO-*d*_6_): *δ* ^13^C-NMR (101 MHz, DMSO-*D*_6_) δ 176.8, 174.6, 168.5, 167.4, 165.8, 165.5, 164.5, 155.4, 150.0, 143.5, 142.4, 135.9, 135. 5, 133.5, 132.4, 130.1, 129.3, 128. 5, 126.3, 123.3, 122.5, 111.3, 76.2, 75.1, 70.3, 66.5, 57.5, 56.2, 53.7, 53.1, 46.8, 43.9, 36.6, 36.4, 32.2; Chemical Formula: C_29_H_29_ClN_6_O_7_S_2;_ LCMS (*m*/*z*): 673.24 [M + H]^+^; Elemental Analysis: [Calculated: C, 51.74; H, 4.34; N, 12.48; S, 9.53; Found: C, 51.79; H, 4.31; N, 12.52; S, 9.58]. IR (KBr, cm^−1^): 3429, 2931, 2858, 1726, 1684, 1526, 1255, 1162.

2-((3*S*,6′*S*,7′*S*,7a′*S*)-7′-(4-chlorophenyl)-5-fluoro-2,2″,4″-trioxo-7′,7a′-dihydro-1′*H*,3′*H*-dispiro[indoline-3,5′-pyrrolo[1,2-*c*]thiazole-6′,5″-thiazolidin]-3″-yl)-*N*-(2-morpholinoethyl)acetamide **5c**

According to the general method, the 5-fluoro-isatin **2c** (82.5 mg, 0.5 mmol) was utilized and the final compound **5c** was obtained as a faint yellow solid compound with 88% chemical yield.

^1^H-NMR (400 Hz, DMSO-*d*_6_): *δ* 11.05 (s, 1H), 8.08 (t, *J* = 5.7 Hz, 1H), 7.45 (q, *J* = 8.5 Hz, 4H), 7.20 (td, *J* = 8.7, 2.8 Hz, 1H), 7.03 (d, *J* = 8.7 Hz, 1H), 6.89 (dd, *J* = 8.7, 4.5 Hz, 1H), 4.83 (q, *J* = 7.8 Hz, 1H), 4.15 (d, *J* = 9.2 Hz, 1H), 4.07–3.91 (m, 2H), 3.81 (d, *J* = 5.9 Hz, 1H), 3.57 (t, *J* = 4.6 Hz, 4H), 3.46 (d, *J* = 6.0 Hz, 1H), 3.17 (dq, *J* = 12.5, 6.7 Hz, 2H), 3.00 (dd, *J* = 9.9, 5.4 Hz, 1H), 2.76 (t, *J* = 8.6 Hz, 1H), 2.34 (dt, *J* = 18.2, 5.7 Hz, 6H); ^13^C-NMR (100 Hz, DMSO-*d*_6_): *δ* 176.2, 174.8, 168.8, 164.7, 159.8, 157.4, 140.1, 135.6, 133.4, 132.3, 129.3, 124.3, 124.3, 118.1, 115.5, 112.0, 76.3, 75.7, 70.0, 66.7, 57.7, 56.2, 53.8, 46.8, 43.9, 36.7, 32.1; Chemical Formula: C_29_H_29_ClFN_5_O_5_S_2;_ LCMS (*m*/*z*): 646.21 [M + H]^+^; Elemental Analysis: [Calculated: C, 53.91; H, 4.52; N, 10.84; S, 9.92; Found: C, 53.90; H, 4.50; N, 10.98; S, 10.02]. IR (KBr, cm^−1^): 3424, 3213, 3085, 2937, 2859, 1712, 1684, 1519, 1239, 1159.

2-((3*S*,6′*S*,7′*S*,7a′*S*)-5-chloro-7′-(4-chlorophenyl)-2,2″,4″-trioxo-7′,7a′-dihydro-1′*H*,3′*H*-dispiro[indoline-3,5′-pyrrolo[1,2-*c*]thiazole-6′,5″-thiazolidin]-3″-yl)-*N*-(2-morpholinoethyl)acetamide **5d**

According to the general method, the 5-chloro-isatin **2d** (90.5 mg, 0.5 mmol) was utilized and the final compound **5d** was obtained as a faint yellow solid compound with 90% chemical yield.

^1^H-NMR (400 Hz, DMSO-*d*_6_): *δ* 11.16 (s, 1H), 8.10 (t, *J* = 5.6 Hz, 1H), 7.51–7.36 (m, 5H), 7.22 (s, 1H), 6.90 (d, *J* = 8.1 Hz, 1H), 4.81 (q, *J* = 7.8 Hz, 1H), 4.15 (d, *J* = 9.1 Hz, 1H), 4.03 (d, *J* = 16.2 Hz, 1H), 3.94 (d, *J* = 16.2 Hz, 1H), 3.78 (d, *J* = 6.0 Hz, 1H), 3.57 (t, *J* = 4.7 Hz, 4H), 3.45 (d, *J* = 6.3 Hz, 1H), 3.17 (dt, *J* = 9.8, 6.5 Hz, 2H), 3.00 (dd, *J* = 9.6, 5.5 Hz, 1H), 2.81–2.72 (m, 1H), 2.34 (dt, *J* = 16.9, 5.6 Hz, 6H); ^13^C-NMR (100 Hz, DMSO-*d*_6_): *δ* ^13^C NMR (101 MHz, DMSO-*D*_6_) δ 175.9, 174.8, 168.7, 164.6, 142.8, 135.6, 133.4, 132.3, 131.5, 131.3, 129.9, 129.3, 127.7, 127.1, 124.6, 112.5, 76.3, 75.5, 70.0, 66.69, 57.7, 56.1, 53.8, 46.8, 43.8, 36.7, 32.2;Chemical Formula: C_29_H_29_Cl_2_N_5_O_5_S_2;_ LCMS (*m*/*z*): 663.16 [M + H]^+^; Elemental Analysis: [Calculated: C, 52.57; H, 4.41; N, 10.57; S, 9.68; Found: C, 52.60; H, 4.39; N, 10.72; S, 9.80]. IR (KBr, cm^−1^): 3423, 3223, 2937, 2859, 1722, 1689, 1525, 1249, 1155.

### 3.4. Crystal Structure Determination of ***5a***

The crystal of **5a** was immersed in cryo-oil, mounted in a loop and measured at a temperature of 120 K. The X-ray diffraction data were collected on a Rigaku Oxford Diffraction Supernova diffractometer using Cu Kα radiation. The CrysAlisPro [47] software package was used for cell refinement and data reduction. An analytical absorption correction (CrysAlisPro [47]) was applied to the intensities before structure solution. The structure was solved by the intrinsic phasing (SHELXT [48]) method. Structural refinement was carried out using SHELXL [49] software with the SHELXLE [50] graphical user interface. The crystal was solved in the space group P2_1_ as an inversion twin. The BASF value was refined to 0.1478. The asymmetric unit contained two independent organic molecules and two water molecules. The ring C19B-C29B-S6B-C21B-N4B was slightly disordered, leading to small positive and negative electron densities near the atom S6B. No disorder model was used in the final refinement. The NH hydrogen atom was located from the difference Fourier map and refined isotropically. The H_2_O hydrogen atoms were also located from the difference Fourier map but constrained to ride on their parent oxygen with U_iso_ = 1.5 U_eq_ (parent atom). All other hydrogen atoms were positioned geometrically and constrained to ride on their parent atoms, with C-H = 0.95–1.00 Å and U_iso_ = 1.2·U_eq_ (parent atom).

### 3.5. Hirshfeld Surface Analysis

The topology analyses were performed using the Crystal Explorer 17.5 program [51].

### 3.6. Computational Methods

All DFT calculations were performed using the Gaussian 09 software package [52] utilizing the B3LYP/6-31G(d,p) method. The geometries were visualized with the GaussView program [53]. Global and local CDFT indices were calculated using the equations given in [37].

## 4. Conclusions

The straightforward synthesis of rhodanine-substituted spiro[pyrrolidine-oxindole]-carboxamide/morpholine derivatives **5a**–**d** using the 32CA reaction methodology was successfully achieved. The supramolecular structure of the studied compound **5a** was analyzed using Hirshfeld calculations. Additionally, the DFT-calculated geometry was found to agree with the reported X-ray structure. The CDFT analysis of the reagents indicated that this 32CA reaction will have a highly polar character due to the supernucleophilic character of AYs **3a**–**d** and the strong electrophilic character of the ethylene derivative **4**, being classified as FEDF. The analysis of the Parr functions suggested that the *meta* reaction paths will be slightly more favorable than the *ortho* ones. However, steric hindrances present along the *meta* reaction paths are responsible for the *meta* regioselectivity experimentally observed. Finally, the MEDT analysis of these 32CA reactions indicated that they take place through a polar non-concerted *two-stage one-step* mechanism associated with the nucleophilic attack of the least substituted carbon of AYs **3a**–**d** on the β-conjugated position of the ethylene derivative **4** as a consequence of the supernucleophilic character of AYs **3a**–**d** and the strong electrophilic character of the ethylene **4**.

## Figures and Tables

**Table 1:** Crystal data of compound **5a**.

	5a
Empirical formula	C_29_H_31_Cl_2_N_5_O_6_S_2_
fw	680.61
Temperature (K)	120(2)
*λ* (Å)	1.54184
Crystal system	Monoclinic
Space group	*P2_1_*
*a* (Å)	14.0397(2)
*b* (Å)	15.9918(2)
*c* (Å)	14.1488(2)
*α* (deg)	90
*β* (deg)	109.439(2)
λ (deg)	90
*V* (Å^3^)	2995.61(8)
*Z*	4
*ρ*_calc_ (Mg/m^3^)	1.509
*μ* (Mo K*α*) (mm^−1^)	3.704
No. of reflns.	18,916
Unique reflns.	9558
Completeness to θ = 67.684°	99.9%
GOOF (*F*^2^)	1.040
*R*_int_	0.0321
*R*_1_ ^a^ (*I* ≥ 2σ)	0.0573
*wR*_2_ ^b^ (*I* ≥ 2σ)	0.1500
CCDC No.	2,104,365

^a^ *R*_1_ = Σ||*F*_o_| − |*F*_c_||/Σ|*F*_o_|. ^b^ *wR*_2_ = [Σ[*w*(*F*_o_^2^ − *F*_c_^2^)^2^]/Σ[*w*(*F*_o_^2^)^2^]]^1/2^.

**Table 2:** Selected bond lengths (Å) and angles (°) for **5a**.

**Atoms**	**Distance**	**Atoms**	**Distance**
Cl1-C16	1.7486	Cl1B-C16B	1.7566
Cl2-C26	1.7396	Cl2B-C26B	1.7446
S5-C10	1.7475	S5B-C10B	1.7685
S5-C11	1.8265	S5B-C11B	1.8185
S6-C20	1.8007	S6B-C20B	1.8247
S6-C21	1.8406	S6B-C21B	1.8196
O1-C3	1.4218	O1B-C3B	1.4399
O1-C2	1.4409	O1B-C2B	1.4117
O2-C7	1.2358	O2B-C7B	1.2247
**Atoms**	**Angle**	**Atoms**	**Angle**
C10-S5-C11	93.42	C10B-S5B-C11B	93.12
C20-S6-C21	90.33	C21B-S6B-C20B	93.33
C3-O1-C2	110.25	C2B-O1B-C3B	109.35
C5-N1-C4	112.35	C5B-N1B-C4B	110.85
C5-N1-C1	112.95	C1B-N1B-C5B	112.04
C4-N1-C1	106.95	C1B-N1B-C4B	108.55
C7-N2-C6	119.66	C7B-N2B-C6B	122.95

**Table 3:** Hydrogen bonds for **5a** (Å and °).

D-H...A	d(D-H)	d(H...A)	d(D...A)	<(DHA)
N2-H2...O1S	0.90(9)	2.09(9)	2.875(8)	146(7)
O2S-H2S...O1B	0.97	1.9	2.843(8)	164
N5-H5...O2	0.78(8)	1.98(9)	2.725(7)	160(10)
O1S-H1SA...O1	0.96	1.93	2.747(8)	140
O1S-H1SB...O5B	0.92	2.03	2.909(7)	159
O2S-H2SA...S6B	0.99	2.77	3.627(6)	146
O2S-H2SA...O5	0.99	2.41	2.910(7)	111
N5B-H5BA...O2B	0.87(8)	2.06(8)	2.870(6)	154(7)
N2B-H2BA...O2S	0.96(9)	1.90(9)	2.845(8)	166(7)
C8-H8A...O4	0.99	2.5	2.848(7)	100
C12-H12...O4	1.00	2.44	2.926(7)	109
C12B-H12B...O4B	1.00	2.46	2.929(7)	108
C8B-H8BB...O4B	0.99	2.49	2.856(7)	102
C14B-H14B...O5B	0.95	2.28	3.143(8)	152
C18-H18...O5	0.95	2.35	3.226(7)	154
C19-H19...O5	1.00	2.43	3.006(7)	116
C21-H21B...O4	0.99	2.49	3.338(8)	144
C28B-H28B...O4B	0.95	2.52	3.154(8)	124

**Table 4:** Important contacts and their interaction distances.

Contact	Distance	Contact	Distance
H3BB…C16B	2.675	O4…H20D	2.585
H6BA…C7	2.678	O3B…H21A	2.464
H1B…C23	2.769	O5B…H1SB	1.974
H1SA…C2	2.382	O4B…H4A	2.542
H15…C10	2.654	O4B…H20A	2.532
H2S…C3B	2.613	O2…H5	1.765
S5B…H21A	2.858	O3…H15	2.595
Cl2B…C24	3.368	O2B…H5BA	1.941
Cl2…C21B	3.44	O3B…H21A	2.464
S6…S5B	3.542	O2S…H2BA	1.856
H2SA…H2BA	2.157	O1B…H2S	1.886
H2B…H1SA	1.989	O1S…H2	1.995
H20B…H5B	2.157	O5…H2SA	2.408
N2…H6BA	2.683	C26B…C17B	3.353

**Table 5:** The calculated bond distances compared with the experimental data ^a^.

Bond ^b^	Calc	Exp	Bond	Calc	Exp
R(1–47)	1.760	1.755	R(15–18)	1.527	1.515
R(2–65)	1.757	1.744	R(21–24)	1.527	1.506
R(3–38)	1.784	1.769	R(27–30)	1.537	1.516
R(3–39)	1.849	1.819	R(33–34)	1.544	1.517
R(4–54)	1.855	1.824	R(37–39)	1.541	1.547
R(4–57)	1.861	1.819	R(39–40)	1.602	1.581
R(5–18)	1.421	1.410	R(39–60)	1.595	1.598
R(5–21)	1.421	1.439	R(40–42)	1.514	1.498
R(6–33)	1.223	1.224	R(40–52)	1.542	1.538
R(7–38)	1.208	1.217	R(42–43)	1.403	1.397
R(8–37)	1.216	1.212	R(42–50)	1.401	1.402
R(9–61)	1.219	1.225	R(43–45)	1.394	1.378
R(10–15)	1.468	1.466	R(45–47)	1.394	1.39
R(10–24)	1.466	1.472	R(47–48)	1.393	1.356
R(10–27)	1.460	1.465	R(48–50)	1.394	1.392
R(11–30)	1.458	1.448	R(52–54)	1.528	1.538
R(11–33)	1.359	1.349	R(60–61)	1.576	1.585
R(12–34)	1.448	1.462	R(60–70)	1.520	1.510
R(12–37)	1.382	1.361	R(62–63)	1.388	1.384
R(12–38)	1.397	1.377	R(62–70)	1.405	1.396
R(13–52)	1.460	1.448	R(63–65)	1.399	1.385
R(13–57)	1.448	1.415	R(65–66)	1.394	1.384
R(13–60)	1.449	1.435	R(66–68)	1.401	1.403
R(14–61)	1.374	1.35	R(68–70)	1.389	1.389

^a^ List of bond angles is given in Table S2 (Supplementary Materials). ^b^ Atom numbering refers to Figure 6.

**Table 6:** Natural charge (NC) population of **5a** ^a^.

Atom	NC	Atom	NC	Atom	NC
Cl 1	−0.0095	H25	0.2085	H49	0.2569
Cl 2	0.0055	H26	0.2373	C50	−0.2050
S 3	0.3188	C27	−0.2699	H51	0.2602
S 4	0.1906	H28	0.2281	C52	−0.0613
O 5	−0.5755	H29	0.2178	H53	0.2461
O 6	−0.6328	C30	−0.2743	C54	−0.5813
O 7	−0.5651	H31	0.2423	H55	0.2644
O 8	−0.5838	H32	0.2559	H56	0.2534
O 9	−0.6000	C33	0.6919	C57	−0.3845
N10	−0.5241	C34	−0.3576	H58	0.2554
N11	−0.6587	H35	0.2708	H59	0.2217
N12	−0.4929	H36	0.2816	C60	0.0656
N13	−0.5118	C37	0.7294	C61	0.6974
N14	−0.6236	C38	0.5354	C62	0.1889
C15	−0.3000	C39	−0.2399	C63	−0.2898
H16	0.2387	C40	−0.2612	H64	0.2620
H17	0.2097	H41	0.2837	C65	−0.0205
C18	−0.1199	C42	−0.0573	C66	−0.2726
H19	0.2354	C43	−0.2169	H67	0.2611
H20	0.2029	H44	0.2618	C68	−0.1894
C21	−0.1199	C45	−0.2447	H69	0.2760
H22	0.2026	H46	0.2573	C70	−0.0911
H23	0.2343	C47	−0.0391	H71	0.4475
C24	−0.2965	C48	−0.2457	H72	0.4196

^a^ Atom numbering refers to Figure 6.

**Table 7:** B3LYP/6-31G(d) electronic chemical potential *μ*, chemical hardness *η*, electrophilicity ω and nucleophilicity *N*, in eV, of AYs **3a**–**d** and **6** and ethylene derivatives **4** and **7**.

	*μ*	*η*	Ω	*N*
AY **3a**	−3.31	3.23	1.69	4.20
AY **3b**	−3.71	3.23	2.14	3.79
AY **3c**	−3.26	3.27	1.62	4.23
AY **3d**	−3.34	3.29	1.70	4.13
AY **6**	−3.11	3.25	1.48	4.39
Ethylene **7**	−4.39	3.78	2.55	2.84
Ethylene **4**	−4.24	3.58	2.51	3.09

## Data Availability

Not applicable.

## Supplementary Materials

The following are available online, Figure S1: Hirshfeld surfaces of **5a**, Figure S2–17: Copy of spectrum data (NMR, IR, MS) for the synthesized compounds **5a–d**; Tables S1–S2: computational data of compound **5a** for the intermolecular interactions and their percentages, also for the bond angles.

## Sample Availability

Samples of the compounds **5a**–**d** are available from the authors.
