# Supplementary material for: Straightforward Regio- and Diastereoselective Synthesis, Molecular Structure, Intermolecular Interactions and Mechanistic Study of Spirooxindole-Engrafted Rhodanine Analogs
Source: Molecules. 2021 Nov 30;26(23):7276. doi: 10.3390/molecules26237276 (PMC8658983; doi:10.3390/molecules26237276)
Supplement: Supplementary file 1 [file molecules-26-07276-s001.zip › molecules-1480366-supplementary.pdf]

SUPPORTING INFORMATION

Straightforward Regio- and Diastereoselective Synthesis, Molecular Structure, Intermolecular Interactions and Mechanistic Study of Spirooxindole-Engrafted Rhodanine Analogs

Assem Barakat,[1,*] Matti Haukka,[2] Saied M. Soliman,[3] M. Ali,[1] Abdullah Mohammed Al-Majid,[1] Ayman El-Faham[3], and Luis R. Domingo[4]

1 Department of Chemistry, College of Science, King Saud University, P. O. Box 2455, Riyadh 11451, Saudi Arabia. maly.c@ksu.edu.sa (M.A.); amajid@ksu.edu.sa (A.M.A.).
2 Department of Chemistry, University of Jyväskylä, P.O. Box 35, FI-40014 Jyväskylä, Finland. matti.o.haukka@jyu.fi (M.H.).
3 Department of Chemistry, Faculty of Science, Alexandria University, P.O. Box 426, Ibrahimia, Alexandria 21321, Egypt. saied1soliman@yahoo.com & saeed.soliman@alexu.edu.eg (S.M.S); ayman.elfaham@alexu.edu.eg (A.E.-F).
4 Department of Organic Chemistry, University of Valencia, Dr. Moliner 50, 46100 Burjassot, Valencia, Spain. domingo@utopia.uv.es
* Correspondence: Email: ambarakat@ksu.edu.sa; Tel.: +966-11467-5901; Fax: +966-11467-5992 (A.B.).

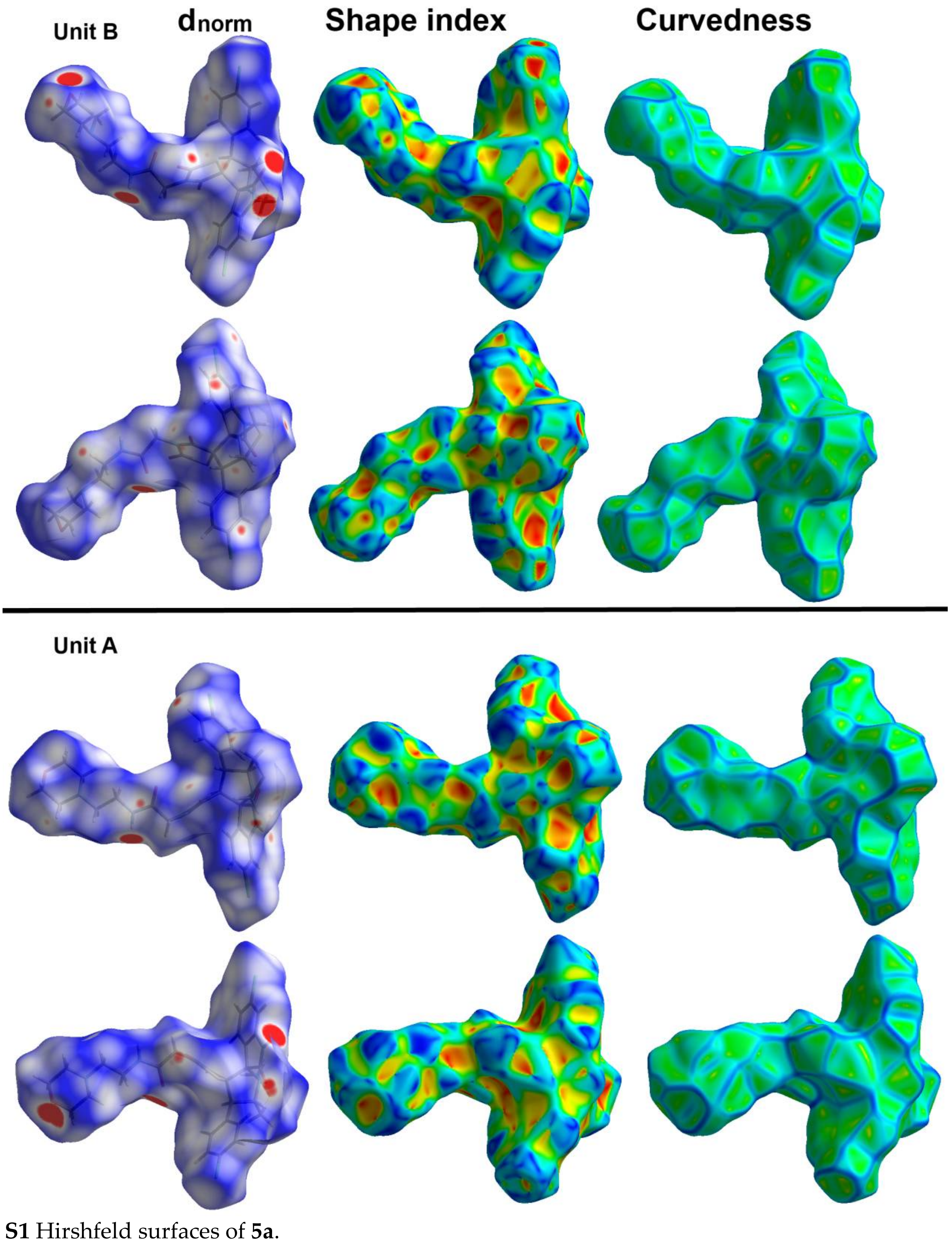

Fig. S1 Hirshfeld surfaces of **5a**.

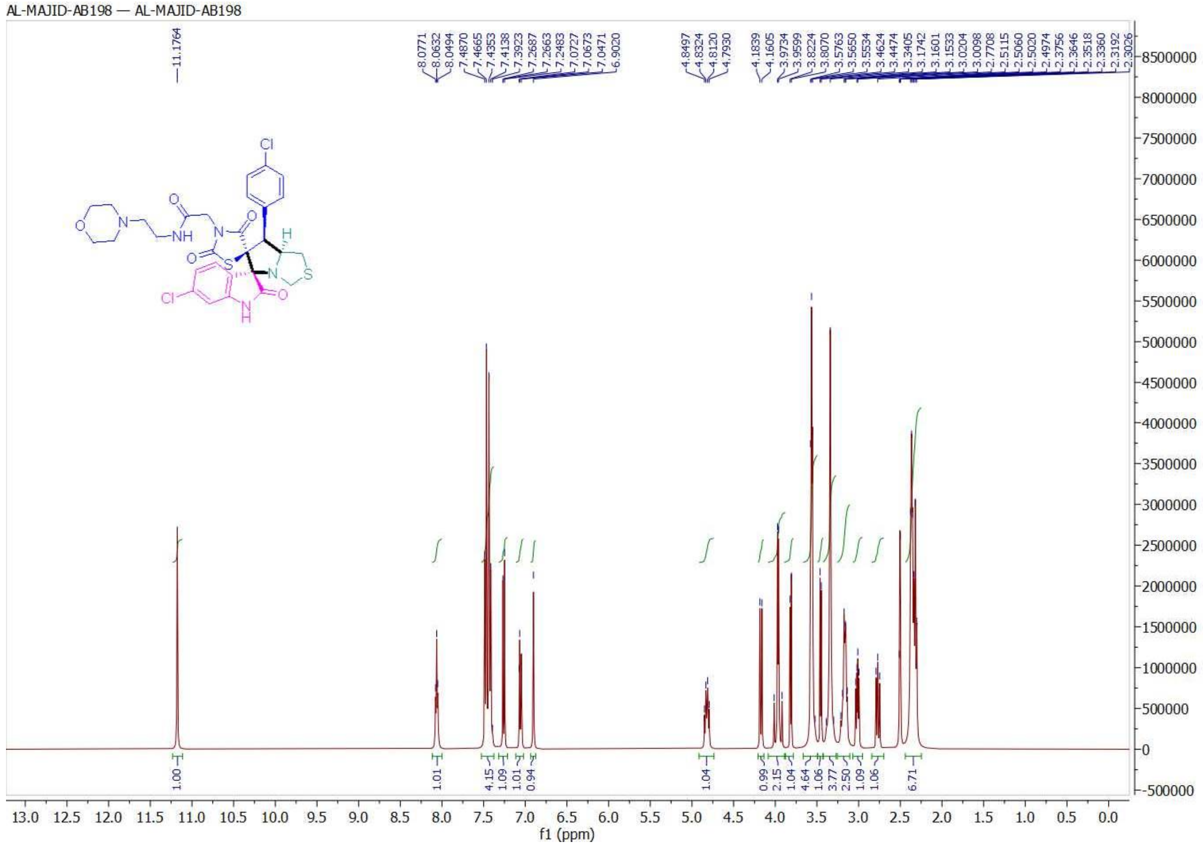

Figure S2: ^{1}H-NMR of **5a**

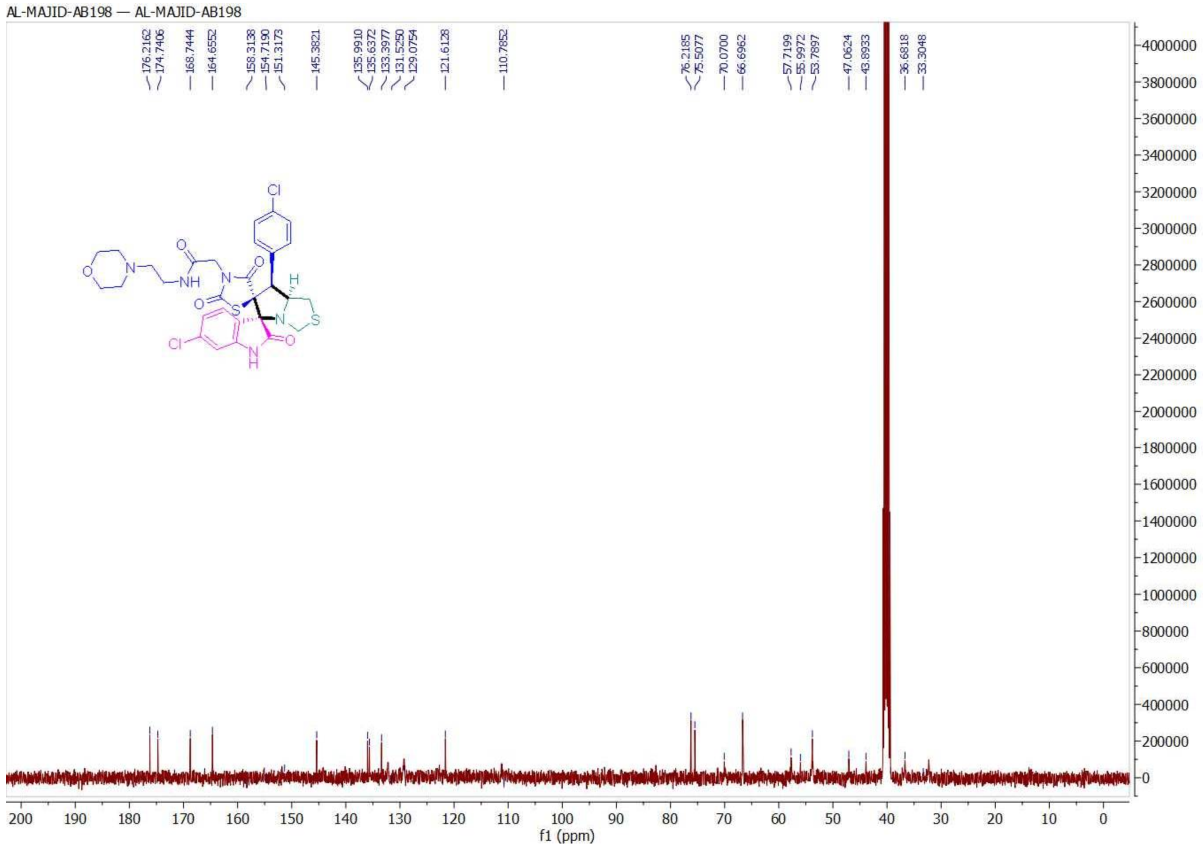

Figure S3: ^{13}C-NMR of **5a**

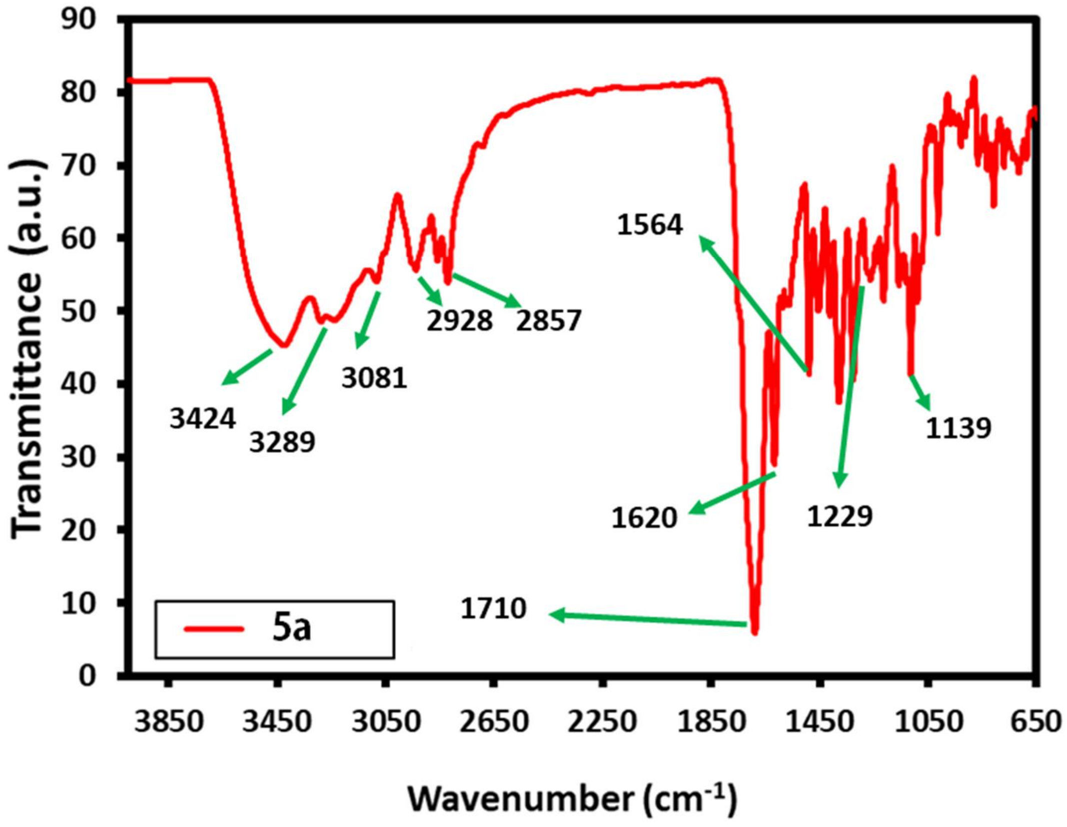

Figure S4: IR of **5a**

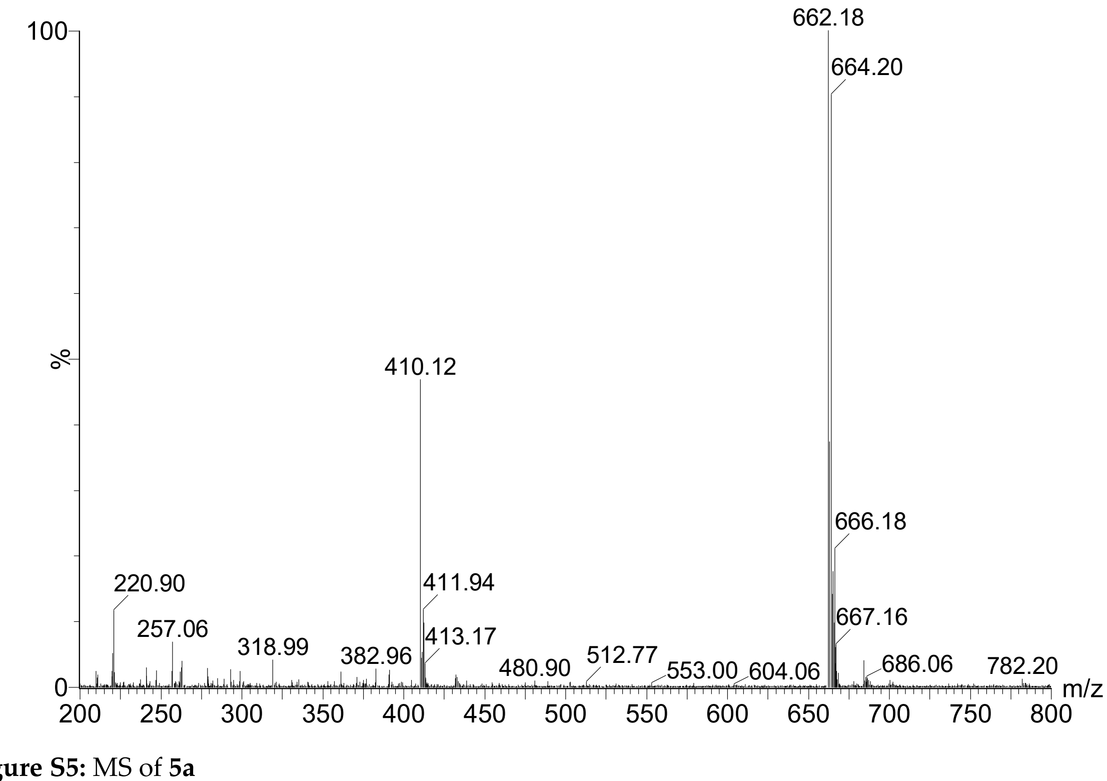

Figure S5: MS of **5a**

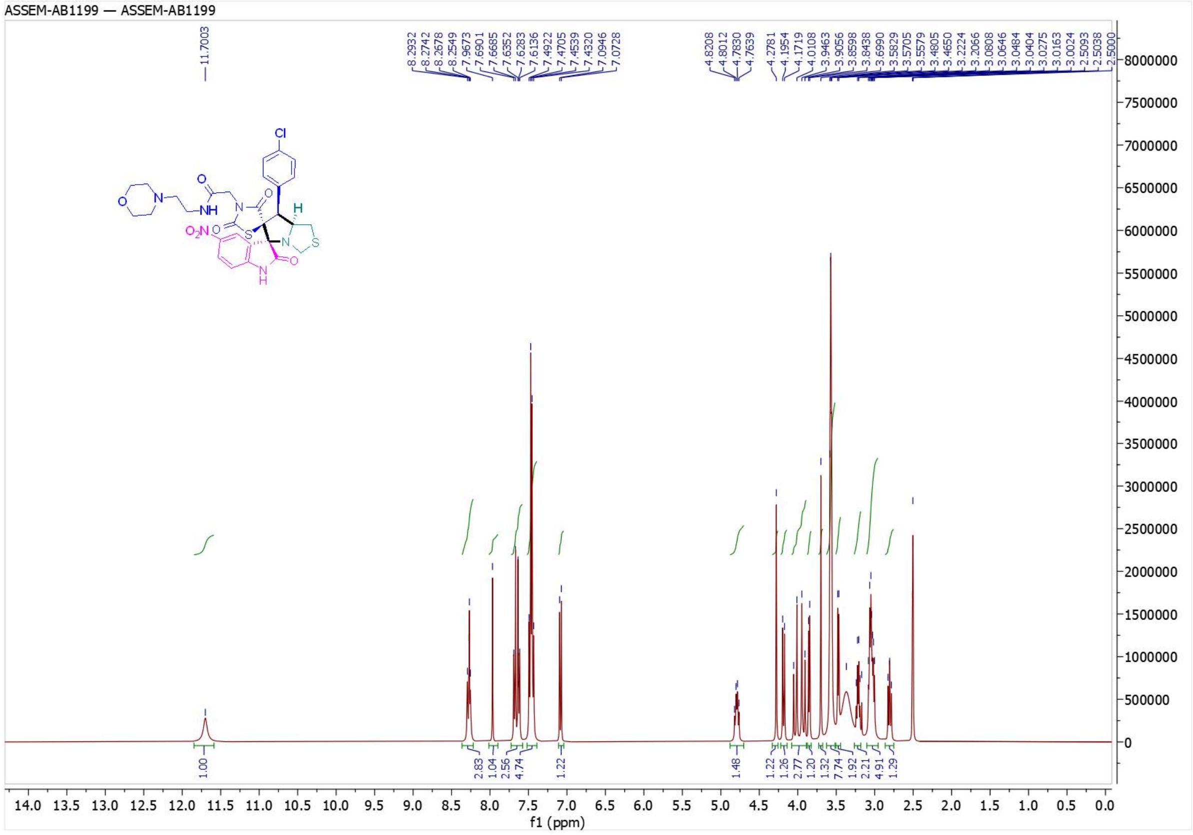

Figure S6: ^{1}H-NMR of **5b**

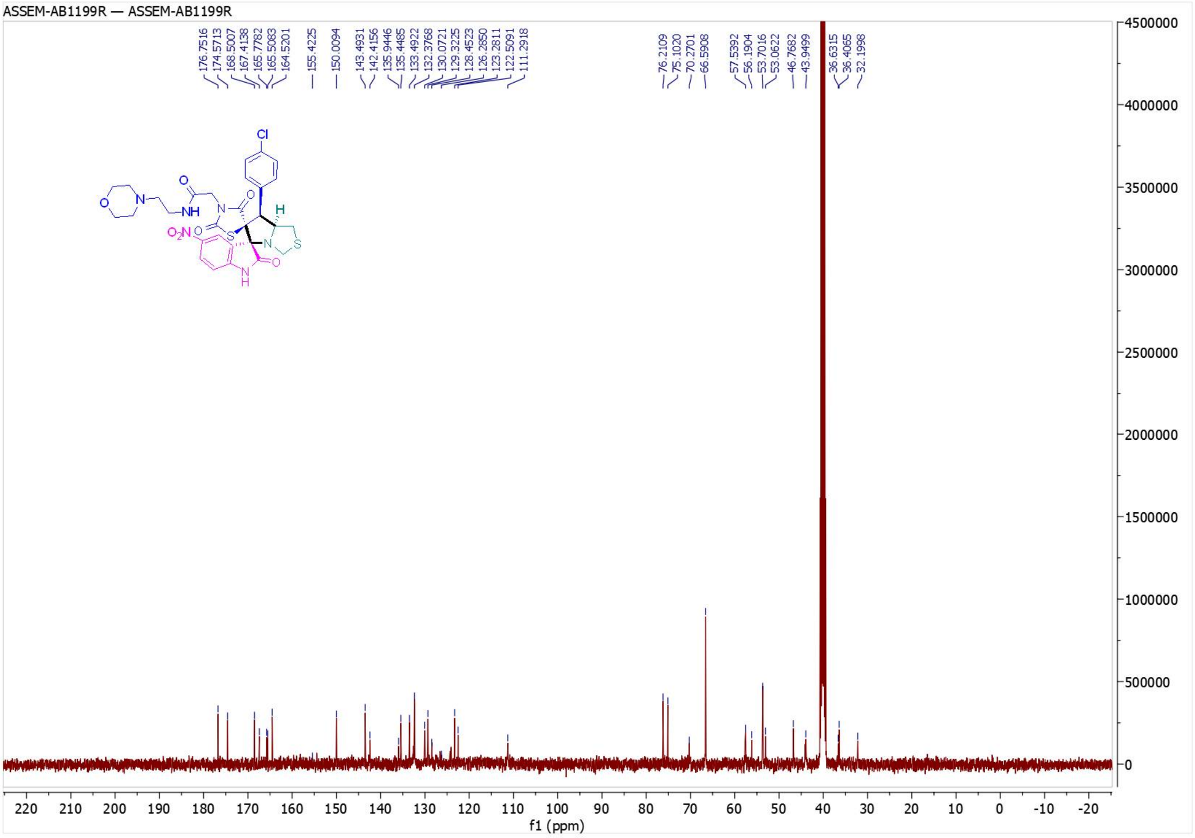

Figure S7: ^{13}C-NMR of **5b**

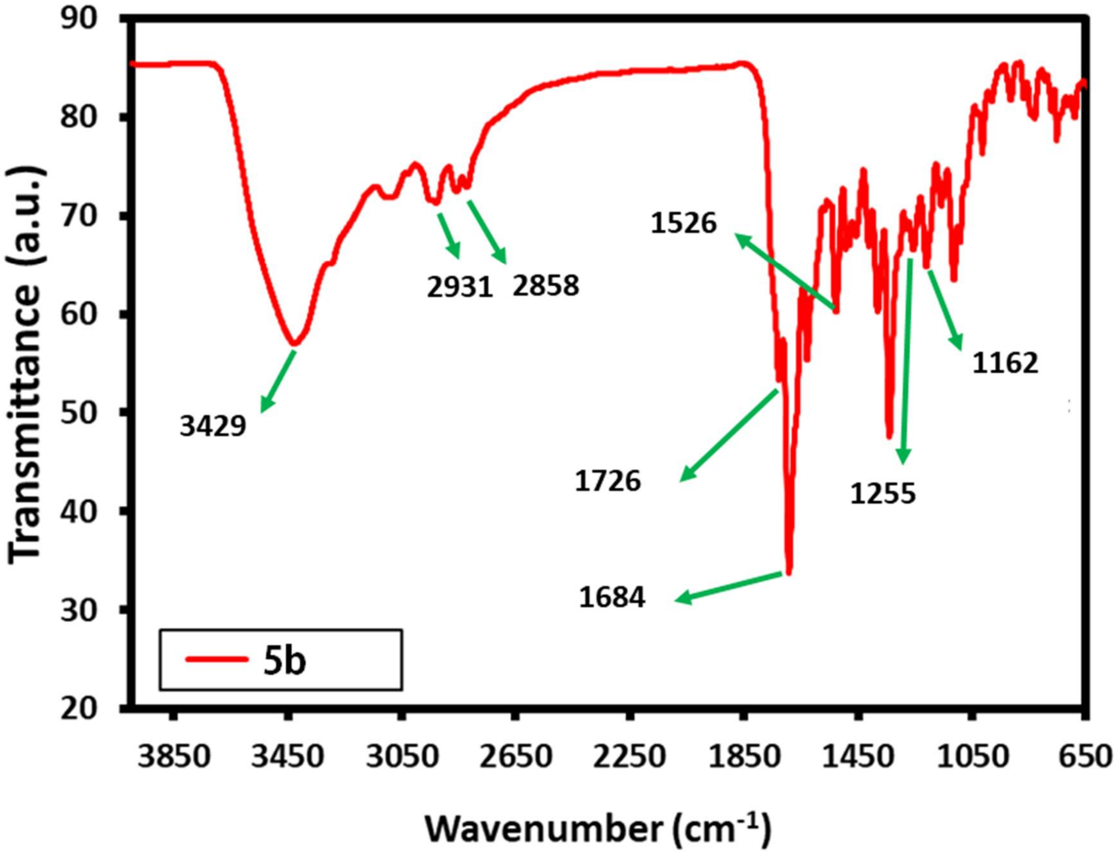

Figure S8: IR of **5b**

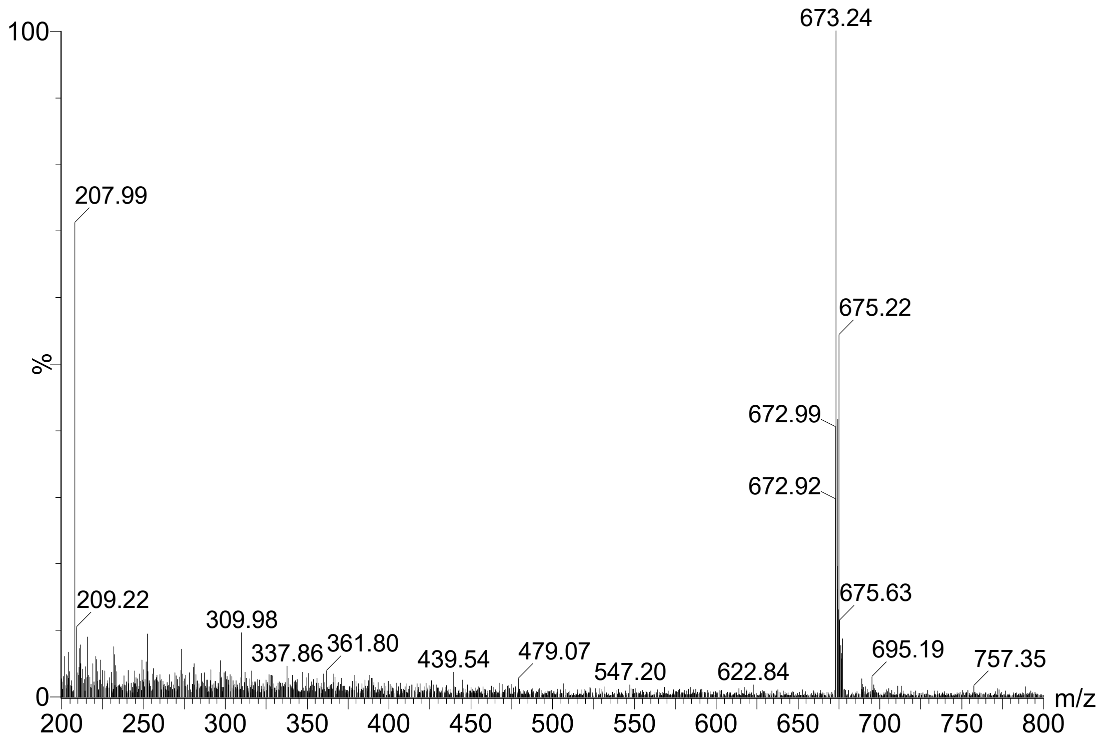

Figure S9: MS of **5b**

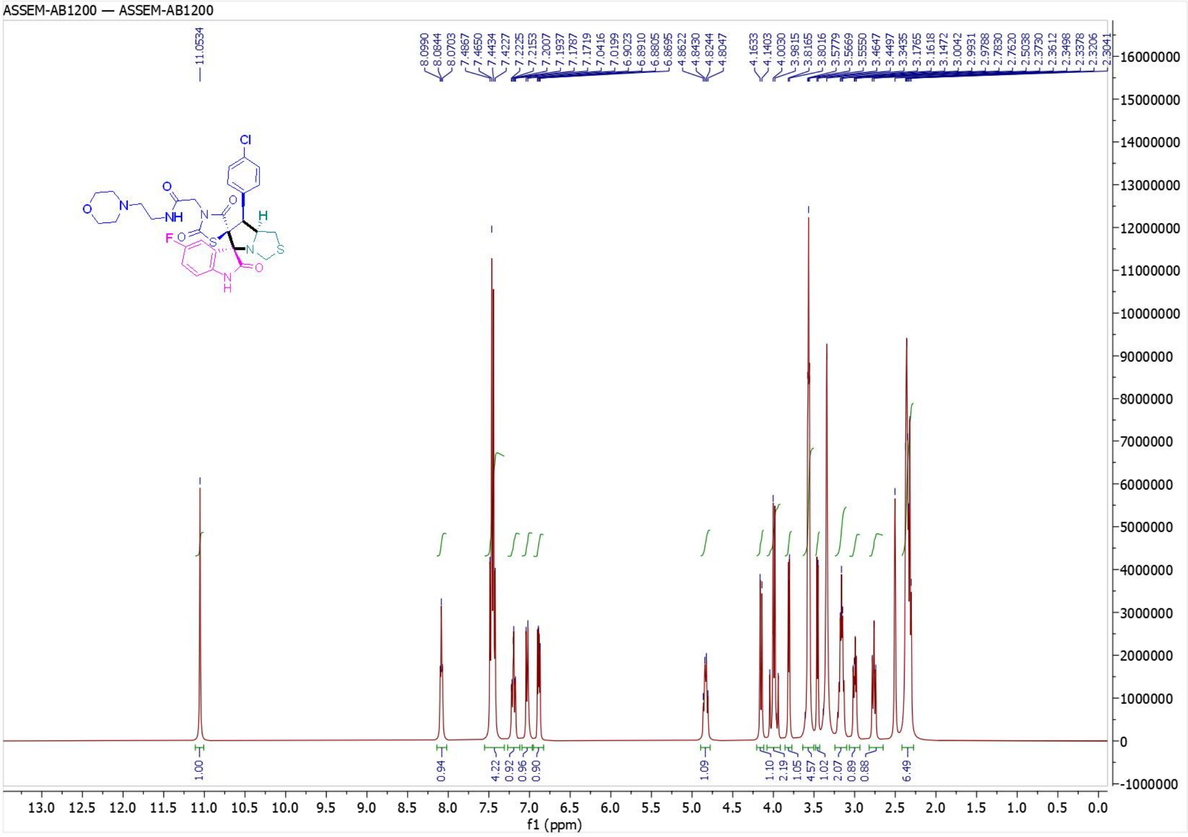

Figure S10: ^{1}H-NMR of **5c**

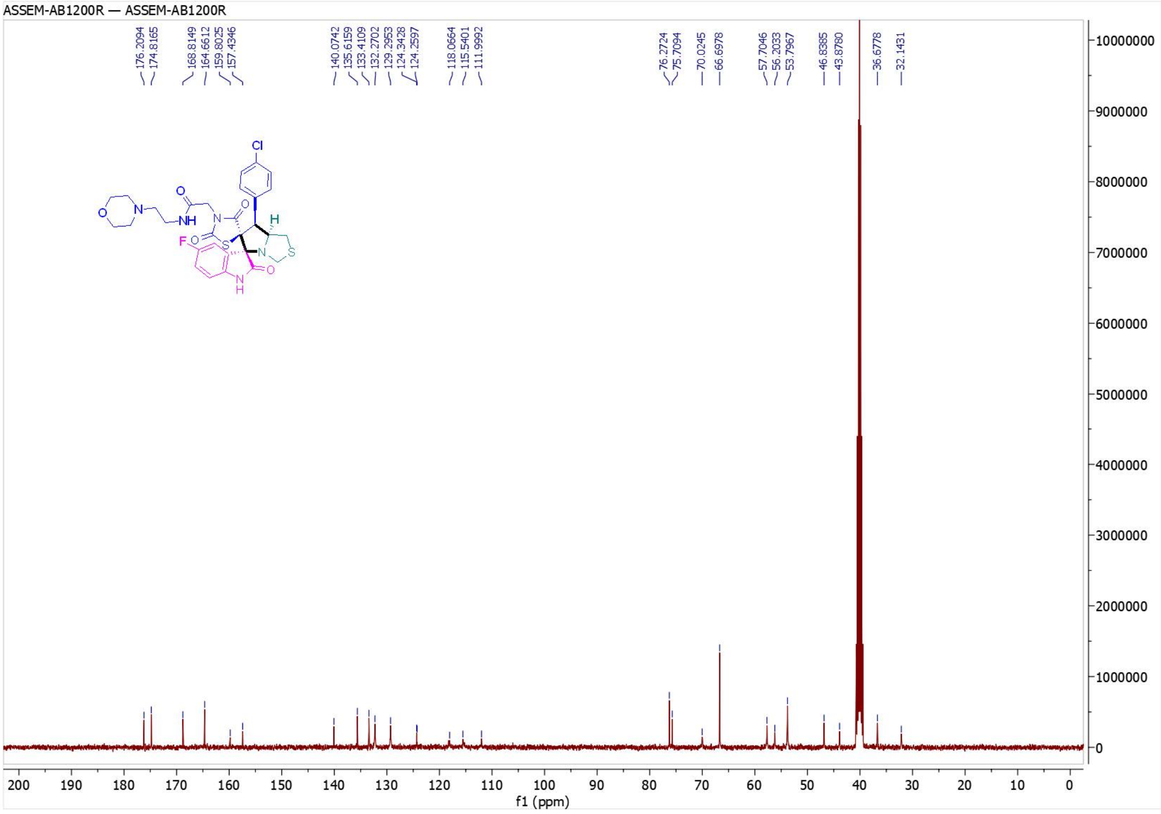

Figure S11: ^{13}C-NMR of **5c**

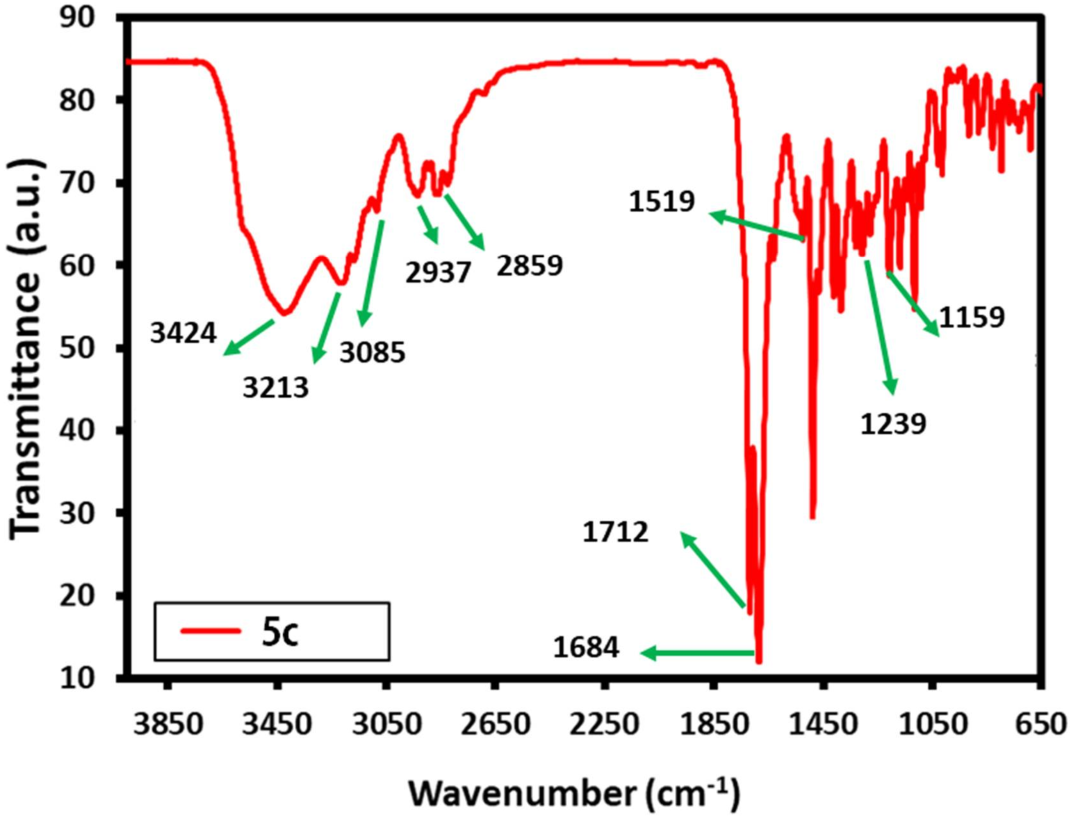

Figure S12: IR of **5c**

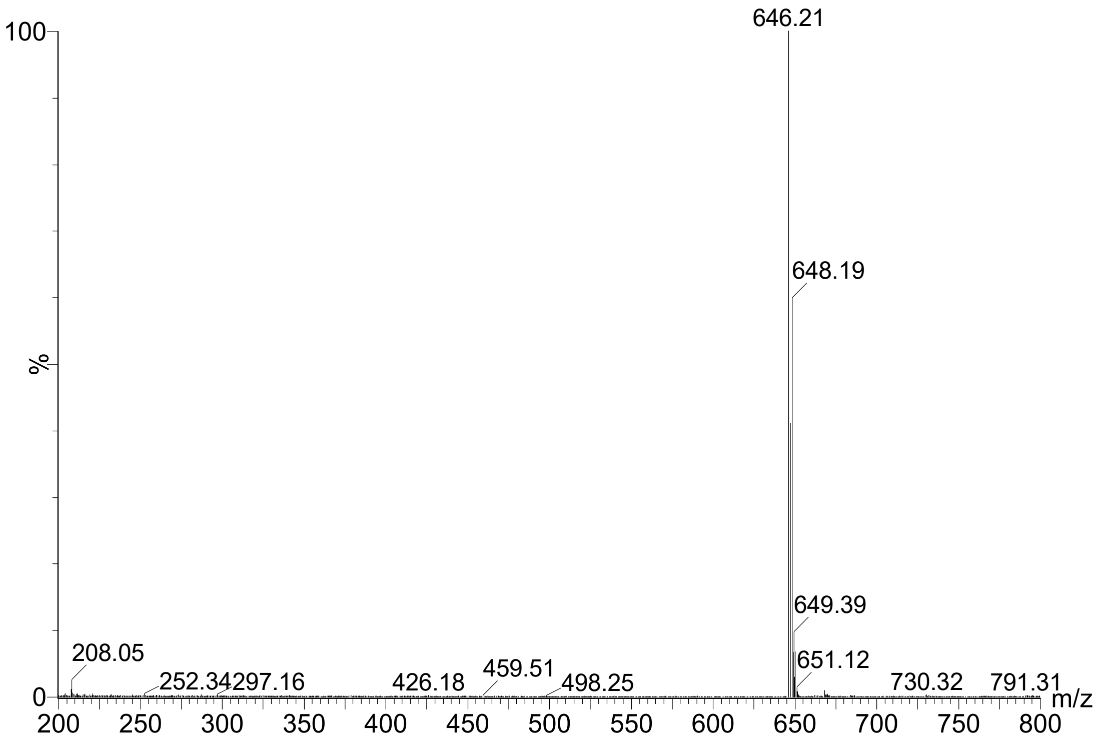

Figure S13: MS of **5c**

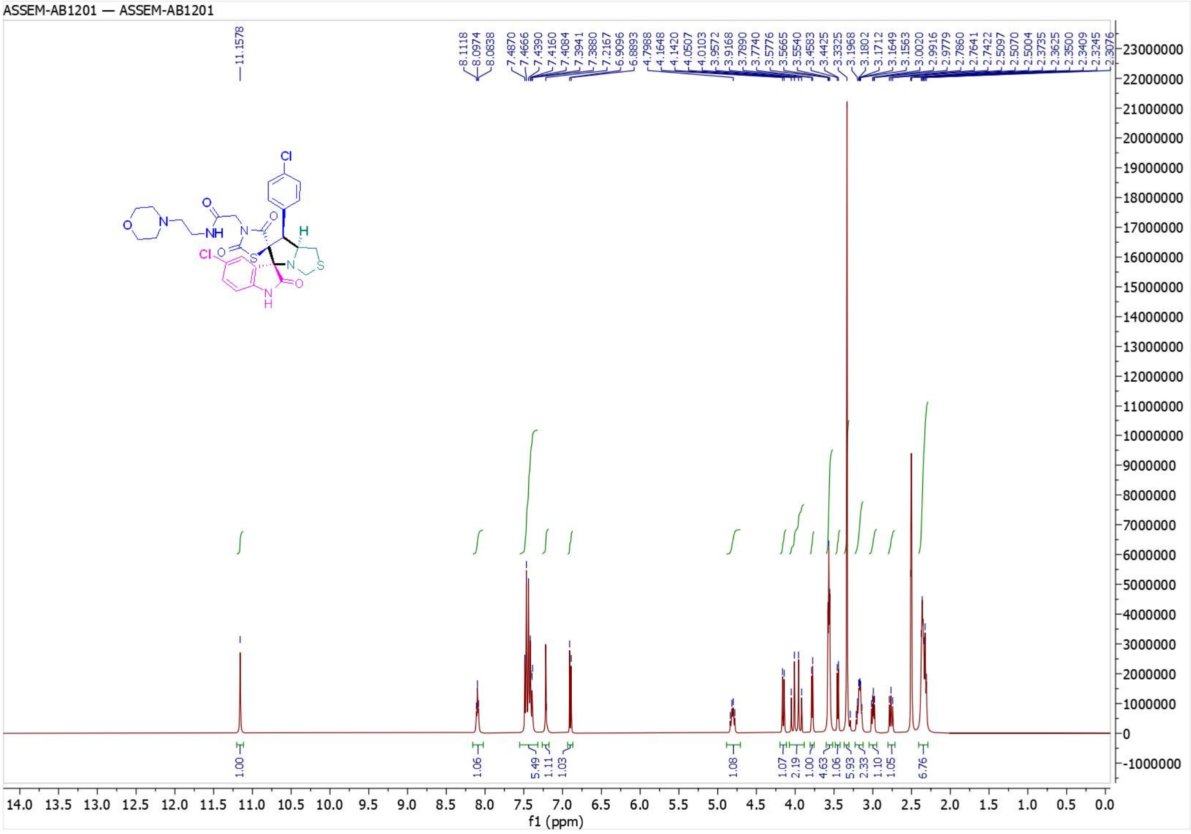

Figure S14: ^{1}H-NMR of **5d**

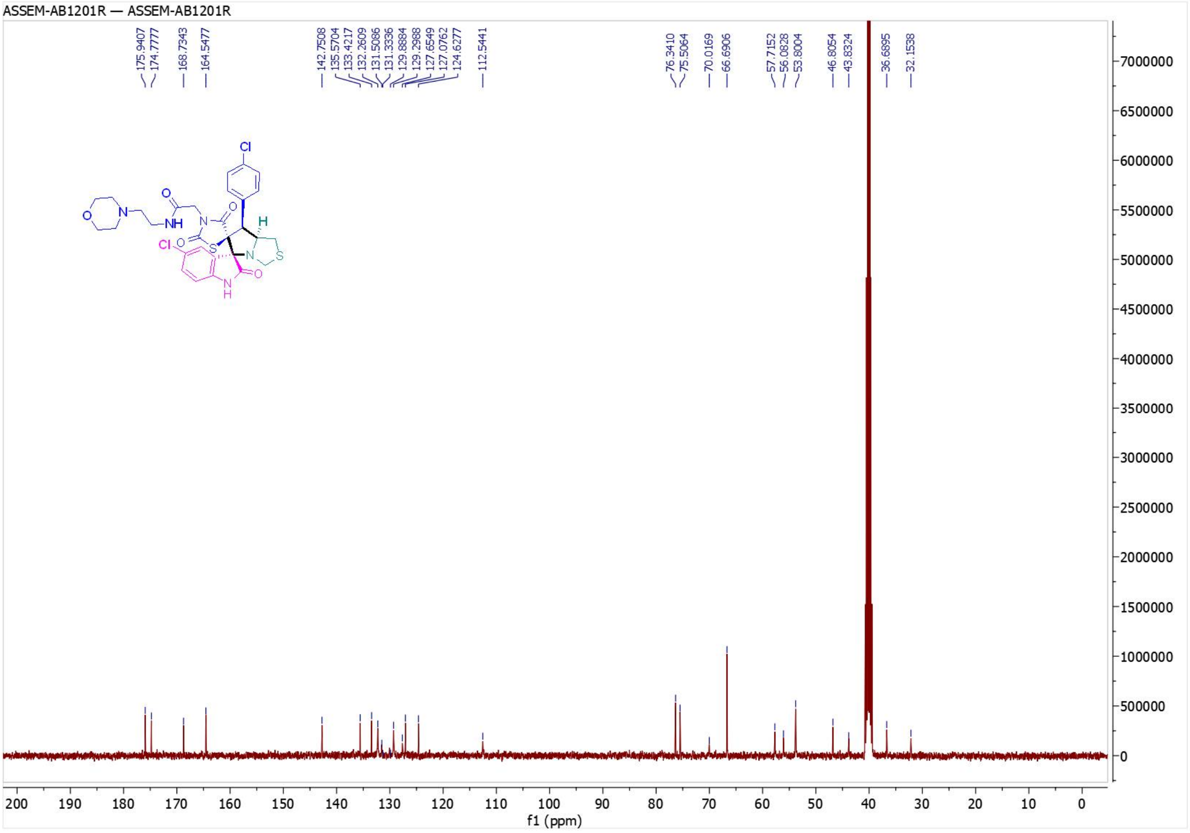

Figure S15: ^{13}C-NMR of **5d**

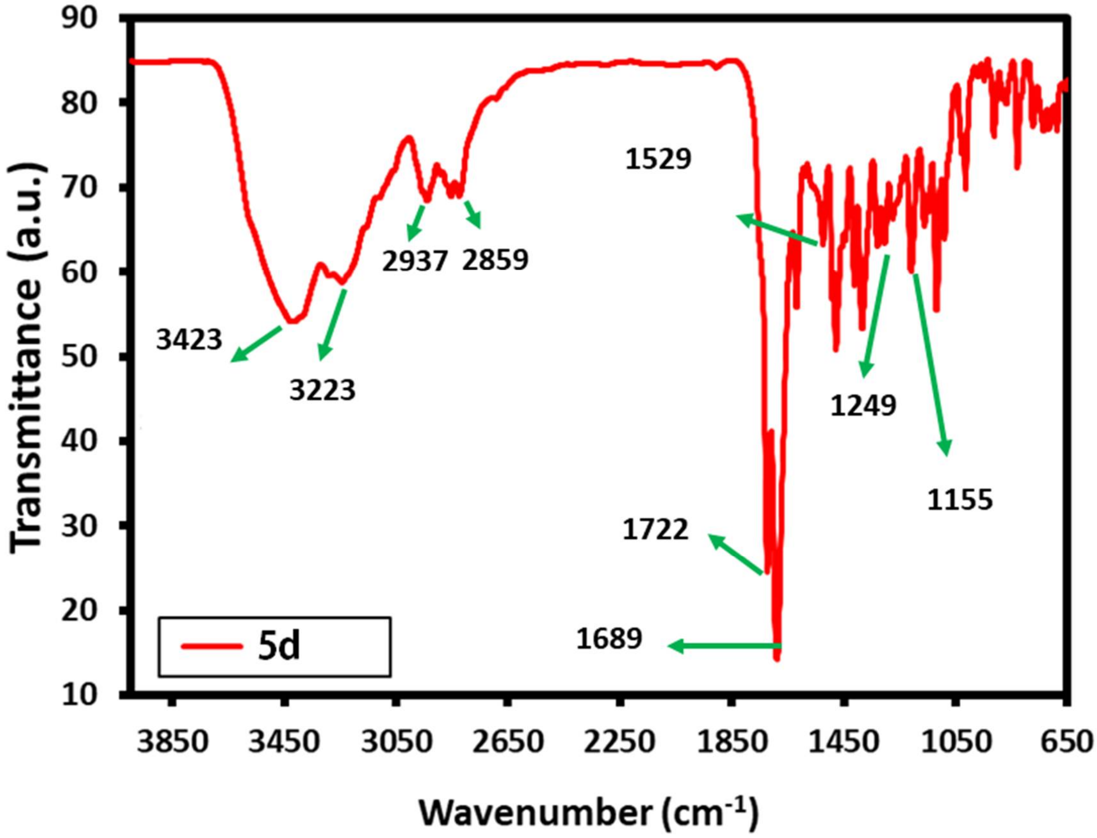

Figure S16: IR of **5d**

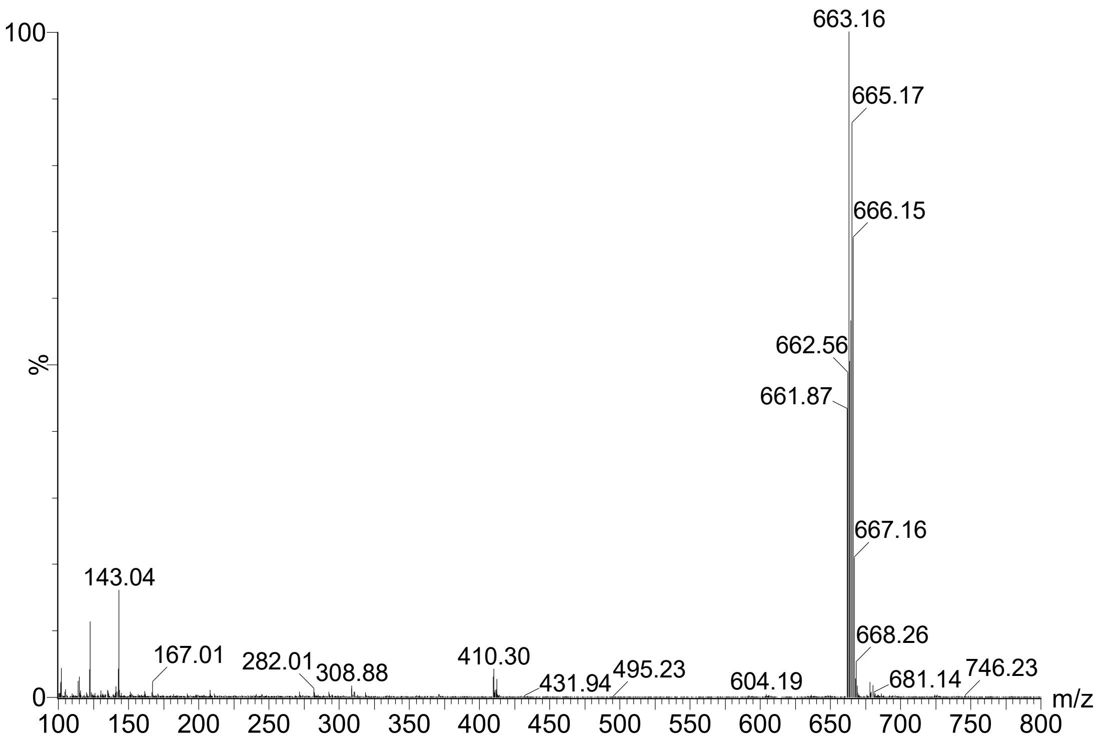

Figure S17: MS of **5d**

Table S1. All intermolecular interactions and their percentages for both molecular units in **5a**.

	**Unit A**	**Unit B**
Cl…Cl	0	0
Cl…N	0.2	0.2
Cl…O	0.2	0.6
Cl…H	14.5	13.1
Cl…C	3.2	3.1
Cl…S	0.5	0.9
S…S	0.7	0.7
S…O	0.8	0.6
S…N	0.2	0.2
S…C	0.1	0.1
S…H	6.4	6.9
O…O	**0.2**	**0.1**
C…O	0.3	0.2
O…H	21.2	19.9
N…H	3.6	2.9
C…C	2.4	2
C…H	8.9	9.7
H…H	36.6	38.8

Table S2 The calculated and experimental bond angles of **5a**[a].

**Parameter**	**Calc**	**Exp**	**Parameter**	**Calc**	**Exp**	**Parameter**	**Calc**	**Exp**
R(1-47)	1.760	1.755	A(1-47-45)	119.5	117.8	A(37-12-38)	117.9	117.5
R(2-65)	1.757	1.744	A(1-47-48)	119.5	120.5	A(12-37-39)	113.0	112.6
R(3-38)	1.784	1.769	A(2-65-63)	118.4	118.0	A(52-13-57)	110.6	111.3
R(3-39)	1.849	1.819	A(2-65-66)	119.3	118.8	A(52-13-60)	109.4	111.3
R(4-54)	1.855	1.824	A(38-3-39)	93.4	93.1	A(13-52-40)	103.2	102.4
R(4-57)	1.861	1.819	A(3-38-7)	125.3	124.6	A(13-52-53)	111.7	111.3
R(5-18)	1.421	1.410	A(3-38-12)	110.3	111.1	A(13-52-54)	104.1	104.3
R(5-21)	1.421	1.439	A(3-39-37)	105.3	105.1	A(57-13-60)	122.1	125.0
R(6-33)	1.223	1.224	A(3-39-40)	116.3	115.3	A(13-57-58)	111.2	110.7
R(7-38)	1.208	1.217	A(3-39-60)	112.0	111.9	A(13-57-59)	113.7	110.7
R(8-37)	1.216	1.212	A(54-4-57)	92.7	93.3	A(13-60-39)	101.1	98.9
R(9-61)	1.219	1.225	A(4-54-52)	104.0	103.6	A(13-60-61)	113.0	116.1
R(10-15)	1.468	1.466	A(4-54-55)	109.1	111.1	A(13-60-70)	114.2	114.4
R(10-24)	1.466	1.472	A(4-54-56)	109.3	111.0	A(61-14-62)	112.3	111.7
R(10-27)	1.460	1.465	A(4-57-13)	103.7	105.4	A(61-14-71)	122.1	122.1
R(11-30)	1.458	1.448	A(4-57-58)	110.1	110.7	A(14-61-60)	107.4	108.2
R(11-33)	1.359	1.349	A(4-57-59)	108.3	110.6	A(62-14-71)	125.6	125.8
R(12-34)	1.448	1.462	A(18-5-21)	110.7	109.4	A(14-62-63)	127.7	126.2
R(12-37)	1.382	1.361	A(5-18-15)	111.4	110.9	A(14-62-70)	109.8	110.2
R(12-38)	1.397	1.377	A(5-18-19)	106.6	109.5	A(16-15-17)	107.6	108.0
R(13-52)	1.460	1.448	A(5-18-20)	110.2	109.5	A(16-15-18)	109.0	109.3
R(13-57)	1.448	1.415	A(5-21-22)	110.2	109.6	A(17-15-18)	109.2	109.2

R(13-60)	1.449	1.435	A(5-21-23)	106.6	109.6	A(15-18-19)	110.5	109.4
R(14-61)	1.374	1.350	A(5-21-24)	111.3	110.1	A(15-18-20)	109.4	109.4
R(14-62)	1.400	1.400	A(6-33-11)	124.1	125.3	A(19-18-20)	108.7	108.0
R(15-18)	1.527	1.515	A(6-33-34)	121.8	121.5	A(22-21-23)	108.7	108.2
R(21-24)	1.527	1.506	A(7-38-12)	124.4	124.3	A(22-21-24)	109.3	109.6
R(27-30)	1.537	1.516	A(8-37-12)	123.0	124.6	A(23-21-24)	110.6	109.7
R(33-34)	1.544	1.517	A(8-37-39)	124.0	122.8	A(21-24-25)	109.0	109.6
R(37-39)	1.541	1.547	A(37-8-69)	101.7	126.5	A(21-24-26)	109.4	109.6
R(39-40)	1.602	1.581	A(9-61-14)	125.7	125.3	A(25-24-26)	107.8	108.1
R(39-60)	1.595	1.598	A(9-61-60)	126.9	127.1	A(28-27-29)	106.8	108.1
R(40-42)	1.514	1.498	A(15-10-24)	109.6	108.5	A(28-27-30)	108.4	109.5
R(40-52)	1.542	1.538	A(15-10-27)	113.0	112.0	A(29-27-30)	109.1	109.6
R(42-43)	1.403	1.397	A(10-15-16)	109.8	109.2	A(27-30-31)	109.4	108.9
R(42-50)	1.401	1.402	A(10-15-17)	111.3	109.2	A(27-30-32)	111.1	108.9
R(43-45)	1.394	1.378	A(10-15-18)	109.8	111.9	A(31-30-32)	108.2	107.7
R(45-47)	1.394	1.390	A(24-10-27)	112.5	110.9	A(33-34-35)	111.2	109.2
R(47-48)	1.393	1.356	A(10-24-21)	109.8	110.4	A(33-34-36)	109.3	109.2
R(48-50)	1.394	1.392	A(10-24-25)	111.9	109.6	A(35-34-36)	109.9	108.0
R(52-54)	1.528	1.538	A(10-24-26)	109.0	109.6	A(37-39-40)	106.8	108.9
R(60-61)	1.576	1.585	A(10-27-28)	107.9	109.6	A(37-39-60)	111.7	110.4
R(60-70)	1.520	1.510	A(10-27-29)	112.7	109.6	A(40-39-60)	104.6	105.2
R(62-63)	1.388	1.384	A(10-27-30)	111.7	110.4	A(39-40-41)	104.5	106.9
R(62-70)	1.405	1.396	A(30-11-33)	122.5	123.0	A(39-40-42)	117.0	117.0
R(63-65)	1.399	1.385	A(30-11-72)	118.3	122.6	A(39-40-52)	103.9	104.2
R(65-66)	1.394	1.384	A(11-30-27)	112.4	113.3	A(39-60-61)	109.2	108.9
R(66-68)	1.401	1.403	A(11-30-31)	109.3	108.9	A(39-60-70)	118.3	118.8
R(68-70)	1.389	1.389	A(11-30-32)	106.3	108.9	A(41-40-42)	107.8	106.8
			A(33-11-72)	118.7	114.2	A(41-40-52)	106.8	106.9
			A(11-33-34)	114.1	113.1	A(42-40-52)	115.9	114.5
			A(34-12-37)	120.6	123.1	A(40-42-43)	122.5	122.2
			A(34-12-38)	119.8	118.9	A(40-42-50)	119.0	120.4
			A(12-34-33)	110.2	111.9	A(40-52-53)	110.4	111.3
			A(12-34-35)	108.3	109.2	A(40-52-54)	118.4	115.7
			A(12-34-36)	107.9	109.2	A(43-42-50)	118.5	117.3

[a]Atom numbering refer to **Fig. 6**
